# Comprehensive review of α-carboline alkaloids: Natural products, updated synthesis, and biological activities

**DOI:** 10.3389/fchem.2022.988327

**Published:** 2022-08-26

**Authors:** Deping Li, Renze Yang, Jun Wu, Bin Zhong, Yan Li

**Affiliations:** ^1^ Department of Pharmacy, First Affiliated Hospital of Gannan Medical University, Ganzhou, China; ^2^ Ganzhou Key Laboratory of Immunotherapeutic Drugs Developing for Childhood Leukemia, First Affiliated Hospital of Gannan Medical University, Ganzhou, China

**Keywords:** α-carboline, neocryptolepine, indolo[2,3-b]quinoline, biological activity, alkaloids

## Abstract

α-carboline (9*H*-pyrido[2,3-*b*]indole), contains a pyridine ring fused with an indole backbone, is a promising scaffold for medicinal chemistry. In recent decades, accumulating evidence shows that α-carboline natural products and their derivatives possess diverse bioactivities. However, hitherto, there is no comprehensive review to systematically summarize this important class of alkaloids. In this perspective, this paper represents the first review to provide a comprehensive description of α-carbolines including natural products, updated literature of synthesis, and their diverse biological activities. Their biological activities including antitumor, anti-microbial, anti-Alzheimer’s disease, anti-atherosclerosis, and antioxidant activities were hilighted. And the targets and the main structure activity relationships (SARs) will be presented. Finally, challenges and future directions of this class of compounds will be discussed. This review will be helpful in understanding and encouraging further exploration for this group of alkaloids.

## 1 Introduction

**FIGURE 1:**
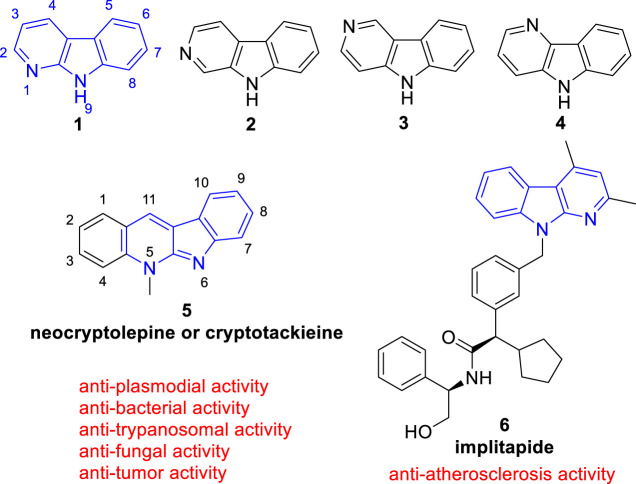
Core structure of simple α- (**1**), β- (**2**), γ- (**3**), and δ-carbolines (**4**). And the representative biological active α-carboline based molecule (**5**) which exists in clinical used plants and (**6**) which reached clinical trails.

Since the structural diversity and wide range of biological activities, carbolines are among the most attractive alkaloids. According to the position of the pyridine nitrogen atom relative to the indole ring, carbolines are classified as α- (**1**), β- (**2**), γ- (**3**), or δ-carbolines (**4**) (Figure 1) (Dai et al., 2018). Among them, α-carboline (9*H*-pyrido[2,3-*b*]indole, **1**), contains a pyridine ring fused with an indole backbone, has attracted renewed attention due to α-carboline based molecules have been successively reported with diverse biological activities including anti-tumor (Pattey and Guyot, 1989), anti-plasmodial (Sharaf et al., 1996), anti-bacterial (Cimanga et al., 1998), anti-fungal (Cimanga et al., 1998), anti-trypanosomal (Jonckerset al., 2002), anti-Alzheimer’s disease (Wang et al., 2017), anti-atherosclerosis (Ueshima et al., 2005), anti-inflammatory (Oda et al., 2009), and neuroprotection activity (Kim et al., 1997).

The classical use of α-carboline alkaloids is that, the African medicinal plant *Cryptolepis sanguinolenta (Lindl.) Schlechter* (*Periplocaceae*), containing a biological active α-carboline based molecule (neocryptolepine or cryptotackieine, **5**), has long been used in the treatment of malaria, amoebiasis, fever, and other infectious diseases (Cimanga et al., 1996; Sharaf et al., 1996) (Figure 1). Nowadays, more and more studies have been conducted to modify the structure of α-carboline natural products or the bare α-carboline scafford to obtain derivatives with better activity. Notebly, implitapide (a molecule containing α-carboline moiety, **6**) has reached clinical trails as an microsomal triglyceride transfer protein (MTP) inhibitor to reduce the progression of atherosclerosis (Ueshima et al., 2005) (Figure 1). Growing bodies of evidence suggests that α-carboline is a promising scaffold in medicinal chemistry for drug discovery.

However, to the best of our knowledge, there is no review on this important class of alkaloids except Wadsworth et al. just reviewed their synthesis in 2015 (Wadsworth et al., 2015). In this perspective, this article aims to provide a comprehensive description of α-carbolines including the natural products, the updated literature of synthesis, and the diverse biological activities of synthetic derivatives, which represents the first comprehensive review of this group of alkaloids. The main contents of this review are as follows: 1) In the first section, α-carboline natural products and their biological activities will be described. 2) Since there was a review of synthesis reported in 2015, updated literature of synthesis will be briefly given in the second section according to the reaction type and the publication date. 3) In the third section, the wide range of biological activities of synthetic α-carboline derivatives will be highlighted according to diseases, targets, research groups, and publication date. In order to better understand the context of the research, literature was first categorized by diseases/targets, then categorized by research groups. In each target/research group section, the logical sequence order was arranged by time. In case where adequate information is available, the structure activity relationships (SARs) of bioactivity will be presented. 4) Finally, challenges and future directions of this class of compounds will be discussed based on our expertise in this field (Liu et al., 2021; Li et al., 2022; Liu et al., 2022; Tian et al., 2022) and carefully analysis of related literature. This work will provide inspiration and encourage further exploration for this group of alkaloids.

## 2 Natural occurring α-carbolines

**FIGURE 2:**
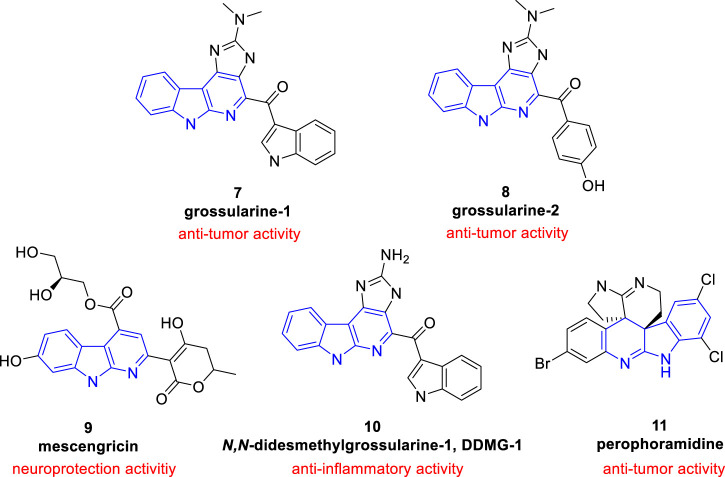
Structures and biological activities of α-carboline based natural products.

In comparison with other classes of carbolines (especially the renowned β-carbolines), α-carbolines are less presented in natural products. Only limited isolated natural products containing α-carboline skeleton were found (Figure 2).

Grossularine-1 (**7**) and grossularine-2 (**8**), isolated from the tunicate *sendrodoa grossularia*, were the first examples of naturally occurring α-carbolines. These two compounds were first found to exhibit striking cytotoxicity toward human and murine tumor cells (Pattey and Guyot, 1989).

Neocryptolepine (also known as Cryptotackieine, **5**) possesses a linear indolo[2, 3-*b*]quinoline system or a chromophore of α-carboline fused with a benzene ring from another point of view, was isolated from the extract of root bark of African medicinal plant *Cryptolepis sanguinolenta* (*Lindl*.) *Schlechter* (Periplocaceae), which is a climbing liana from West and Central Africa used by traditional therapists in the treatment of malaria, amoebiasis, fever, and other infectious diseases (Cimanga et al., 1996; Sharaf et al., 1996). Further research showed **5** exhibited cytotoxcity and anti-plasmodial activity. Thus, as a promising natural product, literature about **5** and its derivatives is emerging (Lavrado et al., 2010; Wang et al., 2019; Nuthakki et al., 2022).

Mescengricin (**9**) was firstly isolated from *streptomyces griseoflavus* 2853-SVS4 as a neuronal cell protecting component (Kim et al., 1997). It possesses an α-carboline skeleton substituted with a hydroxy, a glycerol-ester, and a hydroxydihydropyrone. In the process of screening neuronal cell protecting substances, it was found to protect chick primary mesencephalic neurons from L-glutamatc toxicity with an EC_50_ value of 6.0 nM (Shin et al., 2000).

*N*,*N*-Didesmethylgrossularine-1 (DDMG-1, **10**) was isolated from *Polycarpa aurata* (an Indonesian ascidian). Pharmacological experiment showed that it inhibited the mRNA of TNF-α and IκB-α degradation and inhibited NF-κB binding to DNA site in LPS-stimulated RAW 264.7 cells. Moreover, it also inhibited the production of IL-8. These research suggested that **10** was a promising lead compound to treat chronic inflammatory diseases (Oda et al., 2009).

Perophoramidine (**11**), a polycyclic alkaloid containing an α-carboline moiety, was isolated from *Perophora namei* (a Philippine ascidian)*.* It showed cytotoxicity against HCT116 cell line with an IC_50_ value of 60 µM and induced cells apoptosis *via* poly ADP ribose polymerase (PARP) cleavage within 24 h (Verbitski et al., 2002).

Kapakahines (structures not shown), a large family of cyclic peptides containing an α-carboline skeleton, were isolated from *Cribrochalina olemda* (marine sponge) (Yeung et al., 1996; Nakao et al., 2003). They were often studied as fluorescently labeled chemical probes (Rocha et al., 2015; Kamihira and Nakao, 2021) or anti-malarial agents (Goto et al., 2021).

## 3 General synthetic strategies and advanced synthetic literature of α-carbolines

**FIGURE 3:**
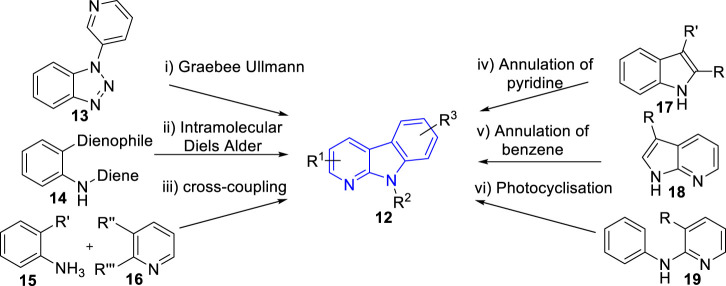
Common synthetic strategies towards α-carbolines (adapted from Wadsworth et al., 2015)

Due to the wide range of biological activities presented by α-carboline natural products and their derivatives, interest in their synthesis has arisen. In 2015, Wadsworth et al. (Wadsworth et al., 2015) summarized the synthetic strategies towards α-carbolines, which including modified Graebee Ullmann method, Diels Alder method, transition metal catalysed of cross-coupling method, annulation of pyridine to indole derivatives method, annulation of benzene ring method, and photocyclisation of anilinopyridines method (Figure 3). As far as we know, there has been no novel strategy for α-carbolines since then on. Here, we just summarized the representative literature on their synthesis since 2015.

### 3.1 Cross-coupling strategy

Using a palladium-catelyzed Ullmann cross-coupling protocol, Yan *et al.* reported a unified approach to obtain the four isomeric carbolines **1**, **2**, **3**, **4** (Yan et al., 2017) (Supplementary Scheme S1). The pivotal steps associated with the unified approach were the palladium-catalyzed Ullmann cross-coupling of 2-iodocyclohex-2-en-1-one (**20**) with the pertinent halogenated nitropyridine (**21, 24, 27, 30**) and the reductive cyclization of the consequential 2-pyridylcyclohex-2-en-1-one (**22, 25, 28, 31**) to give the corresponding 6,7,8,9-tetrahydrocarboline (**23, 26, 29, 32**). Oxidation of these tetrahydro compounds to their fully aromatic analogues (viz., the carbolines **1, 2, 3, 4**) was easily achieveed using 10 wt % palladium on carbon. And this unified approach not only could be used to synthesis core structures of **1, 2, 3, 4**, but also their substituted derivatives, such as harman. While, this protocal was limited by metal catalysis strategy and inflexibility with regard to substituents.

### 3.2 Annulation strategy

Using a transition metal catalysis strategy, Medas and co-workers reported two new methods to afford annulated 2-aryl-α-carboline heterocycles (Medas et al., 2020). The first linear method was described that Rh(I) catalysis was used to form the α-carboline skeleton by [2 + 2 + 2] cyclotrimerization. The second tandem catalytic method stated that using Pd(II) catalyst and mediating a Sonogashira reaction with a [2 + 2 + 2] cyclotrimerization in the same reaction flask to afford the same target molecules (Supplementary Scheme S2).

Through annulation of pyridine ring strategy, Debnath *et al.* reported an α-carbolines synthesis method (Debnath et al., 2021) (Supplementary Scheme S3). Using 2-sulfonamidoindoles (**42**) reaction with acetoxy allenoates (**43**) under phosphine catalysis to afford dihydro-α-carboline and α-carboline scaffolds. At 25°C (room temperature), dihydro-α-carboline structures were achieved exclusively through key reactions of Michael addition, 1,4-proton shift, isomerization, 1,2-proton transfer, phosphine elimination, and aza-Michael addition. At 80°C (higher temperature), α-carboline motifs were achieved *via* key steps of addition-elimination, aza-Claisen rearrangement, tosyl migration, and aromatization.

Although a broad array of strategies for the synthesis of α carbolines were described, many of them are limited by low yields, expensive reagents (such as metal catalysis strategy), starting materials which are difficult to obtain, or inflexibility with regard to substituents. Therefore, novel synthetic strategies of this class of compounds still needs to be explored.

## 4 Biological activities

### 4.1 Antitumor activity

According to the Global Cancer Statistics 2020, cancer is a leading cause of death and an big impediment to increase life expectancy in each country of the world. Worldwide, a predicted 19.3 million new cancer cases and approximated 10.0 million cancer deaths occurred in 2020 (Sung et al., 2021). Undoubtedly, it is imperative to design efficient drugs for the treatment of this disease. Based on its pathogenesis, many targets have been shown to be useful for tumor therapy, which include topoisomerase (Top) (Liang et al., 2019), aurora kinases (Pradhan et al., 2021), breast tumor kinase (Brk) (Tsui and Miller, 2015), microtubule (Kaur et al., 2014) and ras-related protein (RalA) (Fan et al., 2021), etc. Different structures of α-carboline derivatives have been designed and synthesized to regulate these targets.

#### 4.1.1 DNA intercalators/top II inhibitors

**FIGURE 4:**
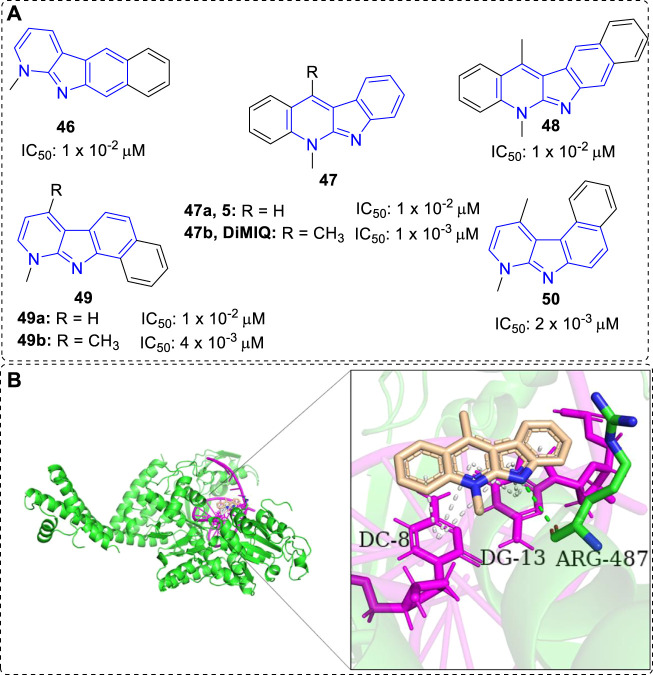
**(A)**Structures and bioactivity of compounds **46–50** with various shapes and sizes. IC_50_ values were tested *in vitro* against KB cells. **(B)** DiMIQ (**47b**) in the active site of Top IIα complexed with DNA (PDB code: 5GWK). **47b** is represented in stick model, carbons in **47b** are colored wheat, DNA is colored magenta, ARG-487 residue of Top IIα is colored green and labeled. Hydrogen bond is presented as green dash line. π-π interactions are presented as white dash lines.

Kaczmarek and co-workers have devoted themselves to the research of α-carbolines since 1870s, but until 1992, they demonstrated 5,11-dimethy-5*H*-indolo[2,3-*b*]quinoline (DiMIQ, **47b**), a synthetic analog of **5**, was a DNA intercalator and Top II inhibitor (Figure 4). It was able to stabilize the Top II-DNA cleavable complex *in vitro* (Pognan et al., 1992). In addition, docking study vividly illustrated that **47b** was intercalated between the base pairs of DNA. There were hydrogen-bonding interaction formed between ARG-487 residue of Top II and indolic N–H of DiMIQ, and π-π interactions formed between DC-8/DG-13 of DNA and aromatic rings of **47b** (Figure 4B). Therefore, indolo[2,3-*b*]quinolines, containing an α-carboline moiety from another point of view, had been proved as a new family of the DNA intercalators and Top II inhibitors. In this regard, the studies of indolo[2,3-*b*]quinolines with anti-tumor activity before and after 1992 would be displayed in this section.

Early in 1988, certain tetra- or pentacyclic benzo-iso-α-carboline system of compounds were synthesized and their anti-tumor properties were evaluated by the research team of Kaczmarek (Kaczmarek et al., 1988a). The results clearly showed that the size and shape of the molecules (**46**–**50**) considerably influenced their bioactivity (Figure 4A). Among them, **47b**, an analog of neocryptolepine (**5**), with a linear, tetracyclic moiety simultaneously bearing two methyl groups at N-5 and C-11 positions was the highest cytotoxicity compound. Further investigations showed it could significantly inhibit tumor growth *in vivo* against mice leukemias P388 and mice melanoma B16. Thus, modification of **47b** has been a hot topic of research due to its anti-tumor activity.

**FIGURE 5:**
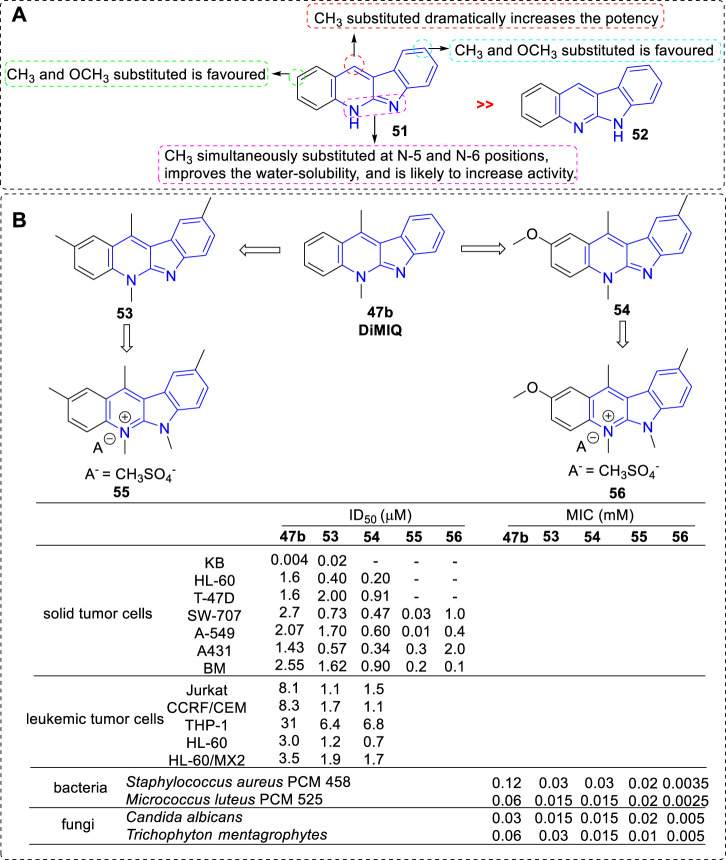
**(A)** SARs of 5*H*- and 6*H*-indolo[2,3-*b*]quinolines; **(B)** Structures and bioactivity of the representative compounds **53**–**56**.

In 1994, the biological activity of 5*H*- and 6*H*-indolo[2,3-*b*]quinolines were compared in the furtherance of SAR study (Czoch et al., 1994) (Figure 5). The results showed that all compounds belonging to the *5H* series (e.g., **51**), i.e., bearing a methyl on the pyridine nitrogen atom, displayed marked cytotoxicity against KB cells with IC_50_ values in the range of 2 × 10^−3^ to 9 × 10^−3^ μM. They stimulated the formation of Top II mediated DNA cleavage at concentration of 0.4–10 μM. While, the compounds belonging to the 6*H* series (e.g., **52**), i.e., lacking a methyl on the pyridine nitrogen atom, were less active in analogous tests compared with the *5H* series. The reason may be that *5H* series were partially protonated at pH value of 7.4 (physiological condition) and at low pH values these compounds occurred in the form of salts, which result in a better water solubility. Among them, the most potent compound was **53** with two methyl groups substituted at C-2 and C-9 positions. Further, other analogs bearing methoxy groups at C-2 and/or C-9 positions were synthesized (Kaczmarek et al., 1999). The cototoxicity were slightly improved in the compounds which bearing a methoxy group, or two methoxy groups at C-2 and C-9 positions compared with that of **47b**. The most potent compound was **54** bearing a methoxy substituted at C-2 position and a methyl substituted at C-9 position. Later, these compounds were tested for their cytotoxicity against a panel of leukemic cell lines, and a subline HL-60/MX2 with reduced expression of Top II (Humeniuk et al., 2002). The results illustrated that all tested compounds possessed cytotoxicity toward these leukemic cell lines and their cytotoxicity relied on the substituents introduced to the indolo[2,3-*b*]quinoline core. Interestingly, THP-1 and HL-60/MX2 cell lines, resistant to etoposide (a reference Top II inhibitor), were susceptible to methoxy- and methyl-substituted derivatives, which suggested that Top II may not be the only target for this class of analogs. In 1998, in order to increase the water solubility, new members of indolo[2,3-*b*]quinoline simultaneously bearing methyl groups at N-5 and N-6 positions were prepared and their cytotoxicity were evaluated (Kaczmarek et al., 1998b). As a result, all obtained derivatives were easily soluble in water with a non-PH-dependent manner, and exhibited cytotoxicity against a penal of tumor cell lines with IC_50_ values range from 0.01 to 0.3 μΜ. They also stimulated the formation of Top II mediated DNA cleavage at concentration of 0.4–0.5 μM. Compound **55**, bearing a methoxy group at C-2 position and a methyl group at C-9 position, showed the most potent activity against A549 cell line.

**FIGURE 6:**
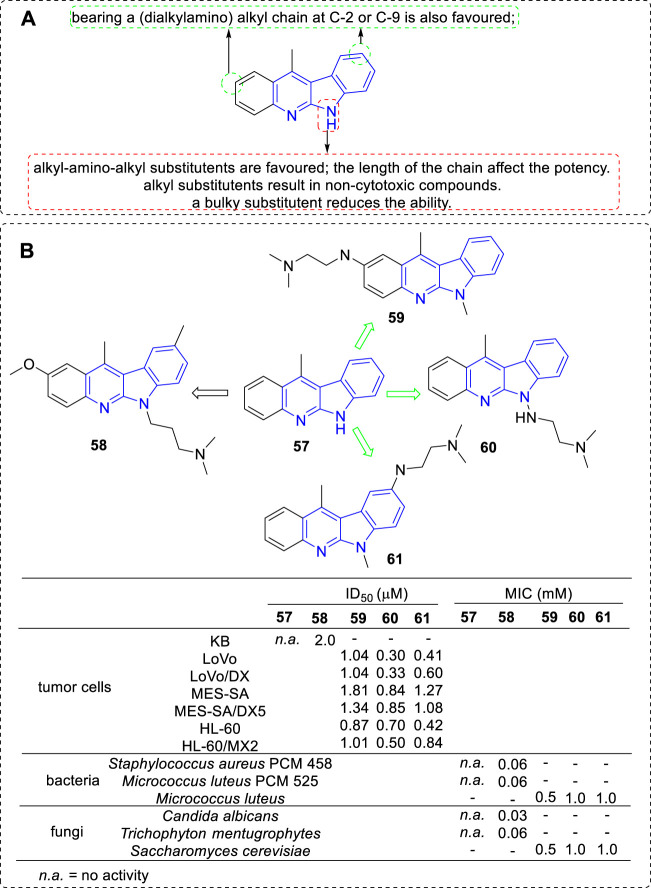
**(A)** SARs of 11-methylated 6*H* series compounds; **(B)** Structures and bioactivity of the representative compounds **57**–**61**.

Although 6*H* series (**52**) were less active than 5*H* series (**51**), further SAR study of 11-methylated 6*H* series (**57**) were still carried out and a panel of derivatives were synthesized in 2002 (Kaczmarek et al., 2002) (Figure 6). The substituents included alkyl-, (alkylamino) alkyl-, and 4-(3-chlorophenyl) piperazi-1-yl-propyl. According to the biological assay, only the introduction of an (alkylamino) alkyl chain into the core structure would be an advisable choice for Top II inhibition, cytotoxic and anti-microbial activity (e.g., **58**). To further explore the effect of the introduction of (alkylamino) alkyl chains, novel derivatives bearing them at C-2 (e.g., **59**), C-9 (e.g., **60**) or N-6 (e.g., **61**) position were synthesized (Godlewska et al., 2005). Their cytotoxic activity against a series of cancer cell lines as well as their drug-resistant sublines were evaluated. All the anologs exhibited DNA binding and Top II inhibiting activity *in vitro*, as a result, showed cytotoxicity against the tested cancer cell lines and constrained the growth of Gram-positive bacteria and fungi. Notably, the results indicated a positive relationship between Top II inhibition and cytotoxicity. Certain compounds possess the ability to inhibit the growth of HL-60/MX2 cell line also suggested that Top II may not be the only target for 6*H* series, which was similar to that of 5*H* series mentioned above.

**FIGURE 7:**
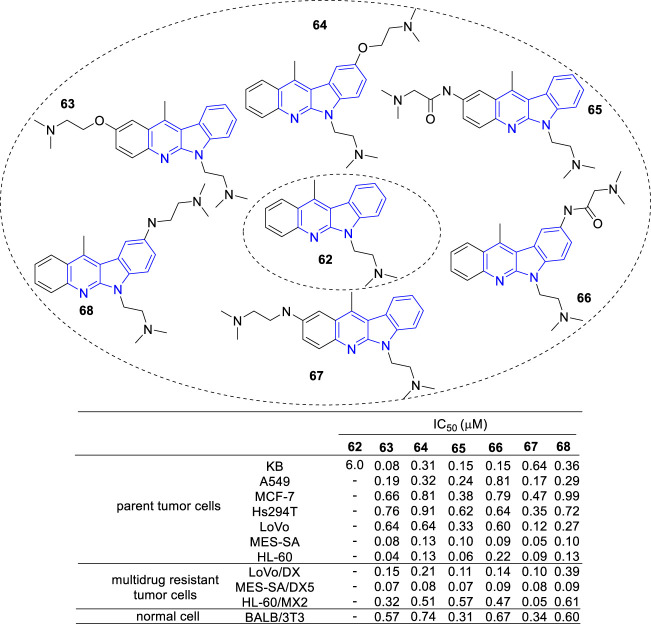
Structure and bioactivity of derivatives (**62**–**68**) which N-6 fixed an (dimethylamino) ethyl chain, C-2 or C-9 linked another (dimethylamino) ethyl chain by amide, ether or amine bond.

Then, novel derivatives which N-6 fixed an (dimethylamino) ethyl chain (**62**), C-2 or C-9 linked another (dimethylamino) ethyl chain by amide, amine or ether bond were synthesized and tested for their cytotoxic activity against a panel of cancer cell lines and multidrug resistant sublines (Luniewski et al., 2012) (Figure 7). Interestingly, all compounds (**63–68**) showed cytotoxic activity against the tested cell lines at a similar level.

**FIGURE 8:**
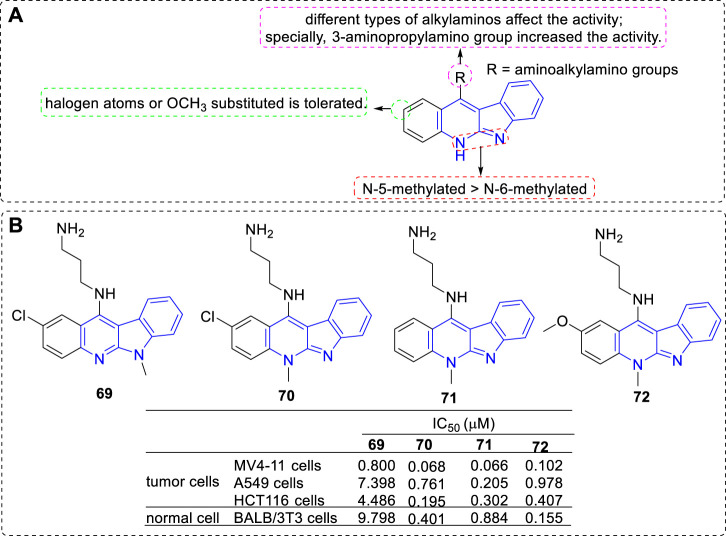
**(A)** SARs of derivatives substituted with aminoalkylamino groups at C-11 position; **(B)** Structures and bioactivity of the representive compounds **69**–**72**.

Further, new derivatives substituted with aminoalkylamino groups at C-11 position were investigated (Wang et al., 2012; Shaban et al., 2017) (Figure 8). Their cytotoxic activity against a penal of cell lines and normal cells were evaluated. The main SARs could be summarized according to the antiproliferative assay: 1) 5-methylated derivatives were more potent than their related 6-methylated derivatives (**69** vs. **70**); 2) A halogen substituent at the 2-position influenced the antiproliferative activity (**70** vs. **71**); 3) An electron-donating group-OMe substituted at C-2 position is tolerated (**71** vs. **72**). 3) Proper alkylamino substitued at C-11 could favourably influence their activities and selectivities, especially the 3-aminopropylamino group (**69–72**).

**FIGURE 9:**
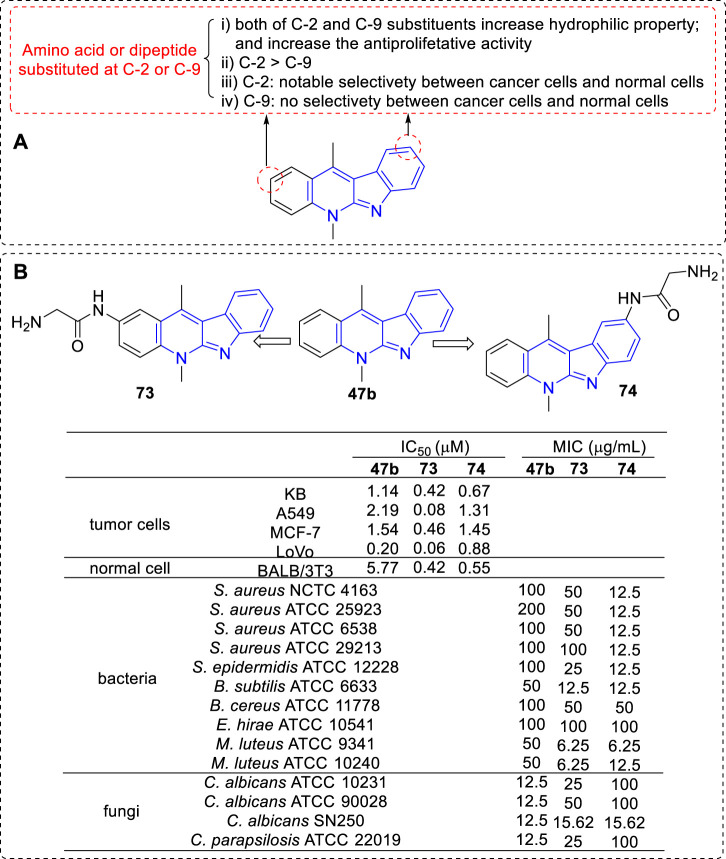
**(A)** SARs of derivatives containing an amino acid or a dipeptide chain at C-2 or C-9 position; **(B)** Structures and bioactivity of the representative compounds **73**–**74**.

To increase selectivity and solubility in water, a series of new analogues of **47b** containing an amino acid or a dipeptide chain at C-2 or C-9 position were synthesized respectively (Sidoryk et al., 2012; Sidoryk et al., 2014) (Figure 9). As a result, all amino acid and peptide derivatives displayed moderate to good antiproliferative activity against A549, KB, MCF-7, and LoVo cell lines. The derivatives attaching a hydrophilic amino acid or a peptide chain to the hydrophobic core of **47b** increased their hydrophilic properties and decreased their hemolytic activity compared to **47b** itself, which was considered to correlate with the low toxicity *in vivo*. Although the author claimed that there were no significant cytotoxic differences between C-2 and C-9 substituted derivatives, we could obtain some key messages by a careful comparison: 1) The toxicity levels of C-2 substituted derivatives was higher than that of C-9 substituted derivatives, especially against A549, MCF-7, and LoVo cell lines (**73** vs. **74**); 2) C-9 substituted derivatives exhibited no significant differences in antiproliferative activity against cancer and normal cell lines; 3) C-2 substituted derivatives showed marked alterations in antiproliferative activity against cancer and normal cell lines, which indicated the presence of a distinct mechanism of action towards these two types of cell lines.

**FIGURE 10:**
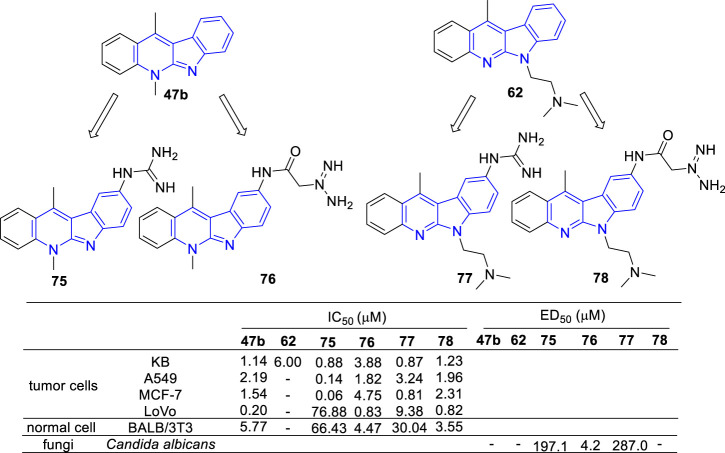
Structures and bioactivity of the representive derivatives (**75**–**78**) which C-9 substitued with guanidine, amino acid or guanylamino acid.

In the continued efforts to improve their solubility properties and selectivity, derivatives containing guanidine or guanylamino acid substituents were manufactured and evaluated for their cytotoxic and anti-fungal activity (Sidoryk et al., 2015; Sidoryk et al., 2017) (Figure 10). As results, most of the tested compounds exhibited cytotoxic activity and compounds **75** and **77**, which guanidine group directly linked to the core of **47b**, exhibited a high selectivity between cancer and normal cells. Although cytotoxic activity was also observed in the *N*-guanylamino acid substituted derivatives (**76** and **78**), no significant selectivity of action was identified. On the contrary, **75** and **77** were inactive against *C. albicans* biofilms, while **76** possessed a potent anti-fungal activity against *C. albicans* biofilms with an IC_50_ value of 4.2 μM. The results indicated that the existence and position of the guanyl moiety in the molecule was essential for cytotoxic activity and selectivity.

**FIGURE 11:**
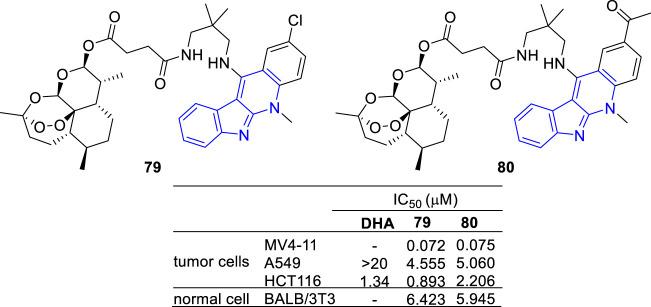
Structures and bioactivity of the representative artemisinin-indoloquinoline hybrids **79** and **80**.

Artemisinin, a sesquiterpene lactone from Artemisia annua, is famous for its use in the treatment of malaria. Besides its anti-malarial activity, artemisinin and its derivatives are identified with anti-tumor potency (Efferth, 2006; Morrissey et al., 2010). With the aim to develop potent and selective antitumor agents, a series of artemisinin-**47b** hybrids were designed and synthesized (Wang et al., 2014). The hybrids **79** and **80** showed an increased antiproliferative activity against A549 and HCT-116 cell lines compared with dihydroartemisinin (DHA) (Figure 11).

**FIGURE 12:**
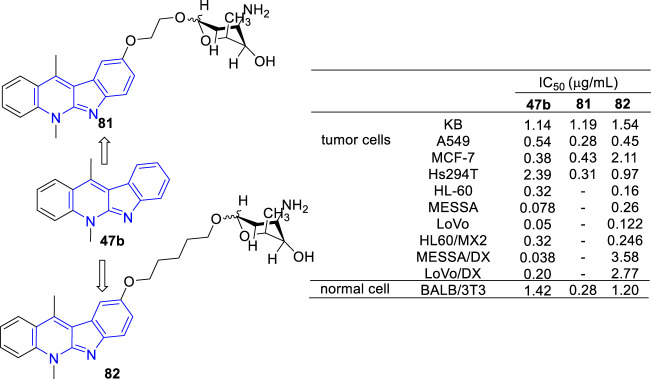
Structures and bioactivity of the representative *O-*aminoglycosides-**47b** hybrids **81** and **82**.

Recently, novel *O-*aminoglycosides-**47b** hybrids were synthesized and evaluated against A549, MCF-7, Hs294T, HL-60, MES-SA, and LoVo cell lines (Figure 12). Hybrids of acosaminyl and **47b** (**81**) showed no selectivity between cancer and normal cells, while hybrids of daunosaminyl and **47b** (**82**) showed good selectivity between cancer and normal cells. Unexpectedly, certain MDR tumor cell lines including LoVo/DX, MES-SA/DX5 were also resistant to these analogs (Bednarek et al., 2006; Roslonek et al., 2016). This was surprising because the parent analogue **47b** displayed antiproliferative activity against all MDR cell lines examined.

Besides the group of Kaczmarek, there were other teams also focused on the research of **47b**. In the screening of novel compounds containing quinoline core as anti-tumor and anti-malarial agents, Akkachairin *et al.* inadvertently found a novel **47b** derivative **83** with moderate antiproliferative ability against a panel of cancer cell lines but alongside high selectivity toward normal cells (Akkachairin et al., 2020) (Supplementary Figure S1).

Altwaijry *et al.* synthesized four derivatives of **47b** (**84–87**) and evaluated their *in vitro* and *in vivo* effect versus Ehrlich ascites carcinoma (EAC). In addition, their antioxidant activity was also tested using the DPPH method. The results indicated that these naturally-based alkaloids exhibited antioxidant activity, notable anti-tumor activity and represented an important class of leads as natural-based antitumor drugs (Altwaijry et al., 2021) (Supplementary Figure S2).

#### 4.1.2 Aurora B kinase inhibitors

Aurora kinases are essential mitotic cell-cycle regulators and play key roles in cell mitosis and division. Among them, Aurora B is a chromosome passenger protein essential for phosphorylation of histone H3, chromosome segregation, and cytokinesis. Aurora B is frequently elevated in cancer, and represents an attractive target for cancer therapy (Carmena and Earnshaw, 2003; Fu et al., 2007; Carmena et al., 2009).

**FIGURE 13:**
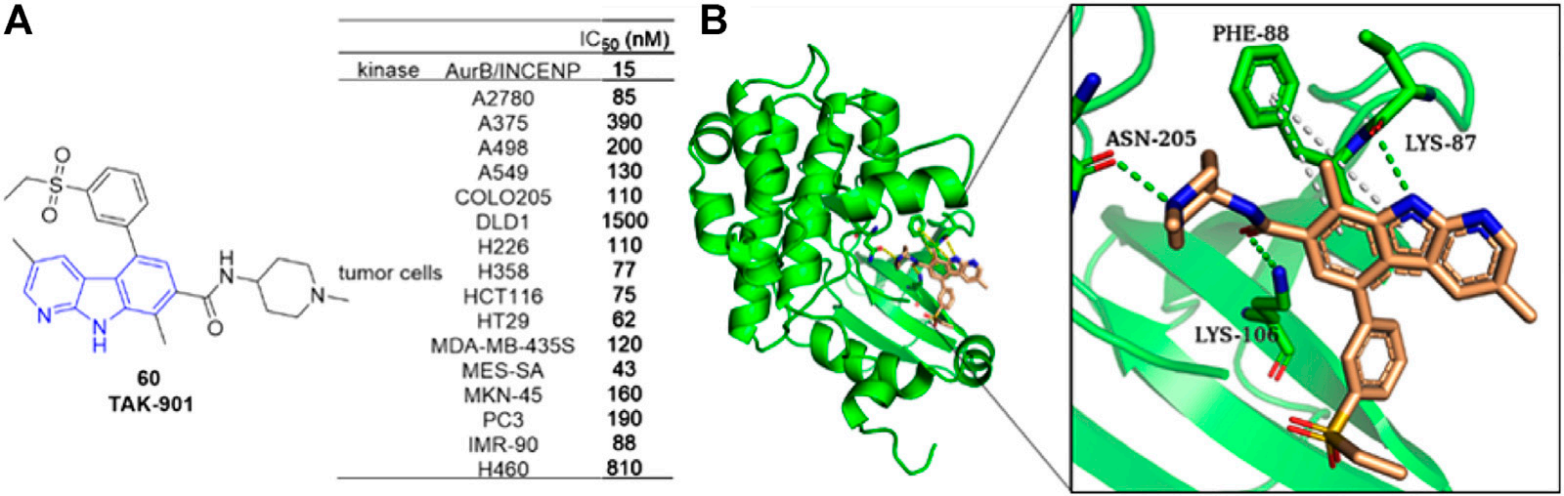
**(A)** Structure and bioactivity of **88** as an Aurora B inhibitor; **(B)** Binding mode of **88** docked into Aurora B kinase (green ribbon representation, PDB code: 4AF3). **88** is represented in stick model, carbons in ligand are colored wheat. Hydrogen bond is presented as green dash lines. π-π interactions are presented as white dash lines.

Farrell et al. reported an Aurora B kinase inhibitor TAK-901 (**88**) (Farrell et al., 2013), which inhibited AurB/INCENP with an IC_50_ value of 15 nM and inhibited various human cancer cell lines with IC_50_ values ranging from 40 to 500 nM. Docking study illustrated that **88** occupied the ATP-binding pocket. Hydrogen-bonding interactions formed between LYS-87, LYS-106 as well as ASN-205 residues and **88**, and π-π interactions formed between PHE-88 residue and α-carboline ring of **88**. Since synthetic difficulty hampered its further clinical evaluation. The team later reported a practical and efficient synthetic process for **88** using an integrated Pd-catalyzed crosscoupling strategy (Mineno et al., 2015) (Figure 13).

#### 4.1.3 Brk inhibitors

Breast tumor kinase (Brk), which was originally found expressed in a metastatic breast tumor, plays an essential role in both cell dysregulation and metastasis. Brk has become an ideal cellular target for tumor therapy because it occurs in a majority of breast tumors but low or undetectable amounts of Brk occurs in normal tissues (Mitchell et al., 1994; Barker et al., 1997; Brauer and Tyner, 2010).

**FIGURE 14:**
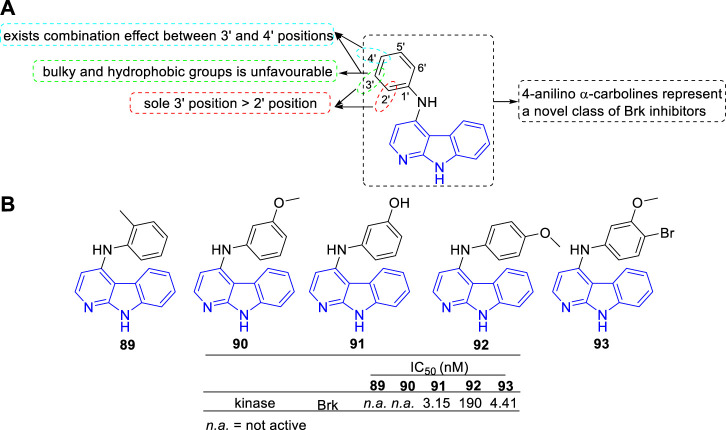
**(A)** SARs of 4-anilino α-carbolines as Brk inhibitors; **(B)** Structures and bioactivity of the representative compounds **89**–**93**.

Mahmoud et al. discovered a series of 4-anilino α-carbolines as a new class of potent Brk inhibitors (Mahmoud et al., 2014). The type and position of the aniline substituents determined the Brk inhibitory activity, which led to IC_50_ values varying from nanomolar to inactive. The main SARs were summarized (Figure 14A): 1) Substituents introduced at 3′-position of the aniline residue seemed more promising compared with substituents introduced at sole 2′-position; 2) At the 3′-position of the aniline residue: A lipophilic thioether function was unfavourable; The bulky and hydrophobic trifluoromethyl substituent showed a slight decrease in Brk inhibition; A chloro substituent and hydroxy substituent with less space showed a strong increase for Brk inhibition; 3) Combined substituent effects showed that 3′-methoxy and 4′-bromo substituents increased the activity compared to sole 3′-methoxy substituent which was found to be inactive (**93** vs. **90**). Structures and bioactivity of the representative compounds (**89**–**93**) were shown in Figure 14B. Later, the team reported two 4-anilino α-carboline derivatives (structures not shown) possessed the ability to induce nonadhesive breast cancer cells death through inhibiting Brk activity (Oelze et al., 2015).

#### 4.1.4 RalA inhibitors

RalA, a member of the Ras small GTPases superfamily, is critical for Ras-mediated human cancer cells proliferation (Hunter et al., 2015; Yan and Theodorescu, 2018). It has been proved that RalA plays an essential role in regulating cancer initiation, invasion, migration, and metastasis, which makes it an interesting tumor therapeutic target (Bum-Erdene et al., 2020; Chen et al., 2020).

**FIGURE 15:**
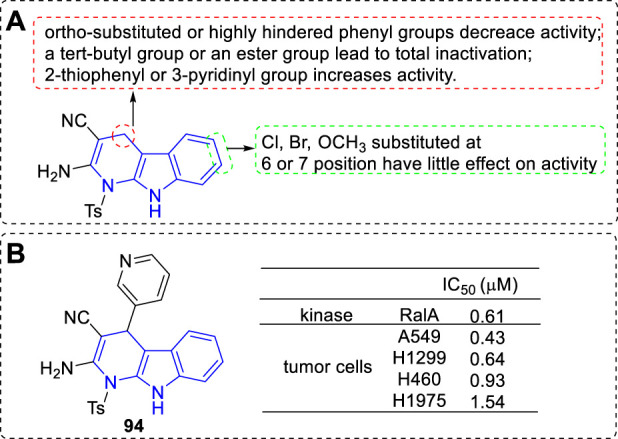
**(A)** SARs of dihydro-α-carboline as RaIA inhibitors; **(B)** Structure and bioactivity of the representative compound **94**.

Leng et al. designed and synthesized a series of dihydro-α-carboline derivatives and some of them could inhibit RalA and proliferative of a panel of cancer cell lines (Leng et al., 2020). The main SARs could be summarized (Figure 15A): 1) C-4 position: 1) The ortho-substituted or highly hindered phenyl residue would lead to lower activity; 2) Introduction of a tert-butyl or an ester group to the phenyl residue would result in total inactivation; 3) 2-thiophenyl or 3-pyridinyl moiety exhibited higher activity; 2) C-6 or C-7 position: The kind and position of the substituents had little effect on the bioactivity. The most potent derivative (**94**) inhibited RalA with an IC_50_ value of 0.61 μM and prolifetative of A549 cell lines with an IC_50_ value of 0.43 μM (Figure 15B).

#### 4.1.5 Microtubule and Top II dual inhibitors

Microtubule consists of microtubulins, participates in the mitotic spindle assembly (Kavallaris, 2010). Top II, a nuclear enzyme, is essential for resolving DNA entanglement and for segregating chromosomes in mitosis (Chen et al., 2013). Both of them are standout anti-tumor targets and their related inhibitors have been extensively used in the treatment of cancer therapy (Jackson et al., 2007; Nitiss, 2009).

**FIGURE 16:**
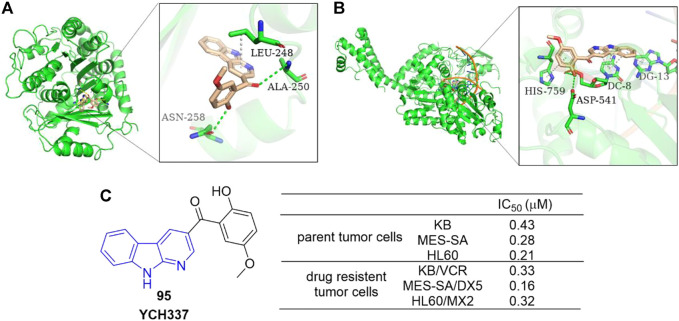
**(A)** Binding mode of **95** docked into microtubule (green ribbon representation, PDB code: 1SA0). **95** is represented in stick model, carbons in **95** are colored wheat. Hydrogen bonds are presented as green dash lines. π-sigma interaction is presented as a white dash line. **(B)** Binding mode of **95** docked into Top IIα (green ribbon representation, PDB code: 5 gwk). **95** is represented in stick model, carbons in **95** are colored wheat. Hydrogen bonds are presented as green dash lines. π-π interactions are presented as white dash lines. **(C)** The structure and bioactivity of the representative compound **95** as microtubule and Top II dual inhibitor.

Yi *et al.* reported an α-carboline derivative YCH337 (**95**), which targeted both microtubule and Top II (Yi et al., 2015). It suppressed microtubule polymerization *via* binding to the colchicine site and subsequently resulted in mitotic arrest. Docking study showed that there were hydrogen-bonding interactions formed between ALA-250 as well as ASN-258 residues of microtubule and **95**, and π-sigma interaction formed between LEU-248 residue and aromatic rings of **95** (Figure 16A). It also inhibited Top II and caused DNA double-strand breaks. Docking study illustrated that there were hydrogen-bonding interactions formed between ASP-541 as well as HIS-759 residues of Top II and **95**, and π-π interactions formed between DC-8/DG-13 of DNA and aromatic rings of **95** (Figure 16B). Its disruption of microtubule was more potent than Top II. Notably, **95** nearly equally inhibited proliferation of MDR tumor cells and their corresponding parent cells (Figure 16C).

#### 4.1.6 ALK inhibitor

The Anaplastic Lymphoma Kinase (ALK) is aberrantly is rearranged or mutated in several tumors including inflammatory myofibroblastic tumor (IMT), anaplastic large-cell lymphoma (ALCL), neuroblastoma, inflammatory myofbroblastic tumor and nonsmall cell lung cancer (NSCLC) patients. (Mologni et al., 2022a). Thus, ALK has become a therapeutic target for personalized medicine in some selected cancers.

**FIGURE 17:**
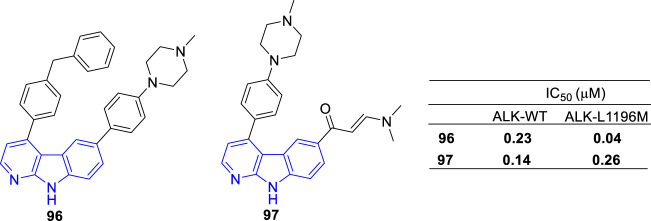
Structure and bioactivity of the representative compounds **96** and **97**.

Mologni and coworkers designed and developmented some novel ALK inhibitors based on a 4,6-substituted α-carboline scaffold. Compound **96** showed potent non-ATP-competitive inhibition of wild-type and mutant ALK in biochemical and cellular assays, as well as in xenograft mouse models (Mologni et al., 2022b). Compound **97** showed selective inhibition of native and mutant drug-refractory ALK kinase *in vitro* as well as in human ALK^+^ lymphoma and in a Ba/F3 model cells (Mologni et al., 2022a; Mologni et al., 2022b) (Figure 17).

#### 4.1.7 Targets indetermination

Early in 1978, Namirski synthesized some 2-position substituted α-carbolines. Among them, compound **98** showed cytostatic activity (Nantka and Kaczmarek, 1978). At 50 mg/kg dose level, it inhibited tumor growth of transplated Ehrlich ascites carcinoma and Nemeth-Kellner lymphoma with inhibition of 59 and 66% respectively. Later in 1986, the team synthesized a series of new α-carbolines and evaluated their anti-tumor activity against L1210 (lymphoid leukemia), P388 (lymphocytic leukemia), and Sarcoma180 (Wieczorek et al., 1986). The results showed that α-carboline derivatives substituted at C-4 position with a methyl group (**99**) or C-6 position with a fluorine (**100**) or chlorine (**101**) atoms caused moderate inhibition of the Sarcoma 180 growth but not other kinds of tumor (Supplementary Figure S3).

**FIGURE 18:**
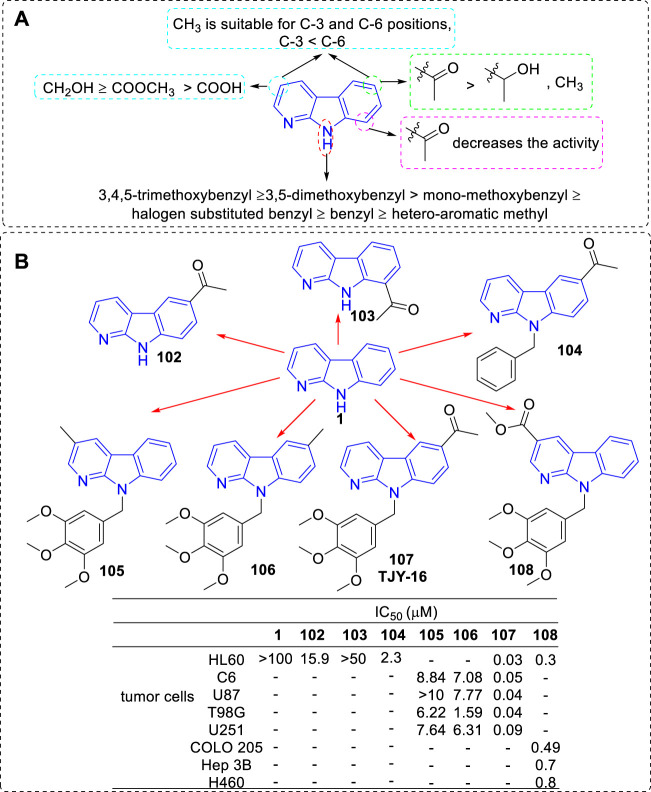
**(A)** SARs of 3, 6, 8, or 9-substituted α-carbolines for their antitumor activity; **(B)** Structures and bioactivity of the representative compounds **102**–**108**.

In 2010 and 2016, the team of Li successively reported a series of 3, 6, 8, or 9-substituted α-carbolines and tested their antitumor activity (Tsai et al., 2010; Huang et al., 2016; Lin et al., 2016). Based on the results of activity, the main SARs could be summarized (Figure 18A): 1) The nonsubstituted α-carboline (**1**) had almost no cytotoxicity; 2) An N-9 methylaryl moiety was a critical functional moiety for maintaining the potency. The following rank order was found: 3,4,5-trimethoxybenzyl ≥ 3,5-dimethoxybenzyl > mono-methoxybenzyl ≥ halogen substituted benzyl ≥ benzyl ≥ hetero-aromatic methyl. 3) Based on the C-3 substituents, the following rank order was found: CH_2_OH ≥ COOCH_3_ ≥ COOH. 4) Introduction of an acetyl group at C-6 but not C-8 position increased the inhibitory activity; 5) Substituents of C-3, C-6, and C-9 showed synergistic effects. The structures of the representative compounds **102–108** and their activity were shown in Figure 18B.

Besides, Zhang *et al.* achieved some 2 or 2,4-substituted α-carbolines with moderate anti-tumor activities *via* an effective and convenient method (Zhang et al., 2014). Since a few of these compounds were selected to determine their anti-tumor activity, the SAR could not be obtained. The most potent compound **109** exhibited antiproliferative of BEL-7402 cells with an IC50 value of 0.58 µM (Supplementary Figure S4). Emam et al. obtained several Copper (II) complexes with aminoalkylaminoneocryptolepine as anticancer agents (Emam et al., 2015). The most potent compound (structures not shown) exhibited antiproliferative activity of HT-29 cells with an IC50 value of 0.58 µM.

### 4.2 Anti-microbial activity

#### 4.2.1 Anti-plasmodial activity

Due to the introduction of the artemisinin-based combination therapies (ACTs), a great success has been achieved globally in the treatment of malaria over the period 2000 to 2019, but there were still an estimated 229 million malaria cases in 2019 (https://www.who.int/teams/global-malaria-programme). Therefore, the development of novel drugs to treat malaria is still needed. Natural products isolated from plants are an important resource for the discovery of new drugs. Compound **5**, isolated from the root bark of African plants *Cryptolepis sanguinolenta*, is one of the representative natural products with anti-plasmodial activity besides artemisinin. Compound **5** and its derivatives have achieved great attention for their ability against malaria.

**FIGURE 19:**
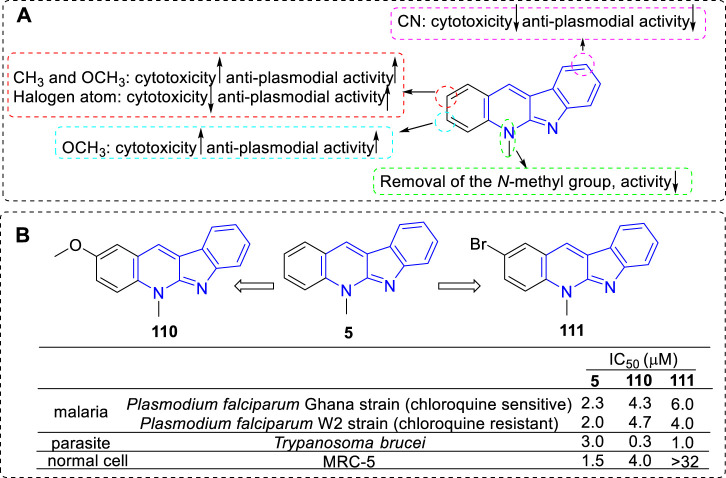
**(A)** SARs of **5** derivatives for their anti-plasmodial activity; **(B)** Structures and bioactivity of the representative compounds **110** and **111**.

Pieters and coworkers have devoted themselves to the research of compound **5** and its derivatives against malaria since the 1990s (Cimanga et al., 1997; Cimanga et al., 1998). But until in 2002, they reported a set of synthetic analogs and evaluated their anti-plasmodial activity against chloroquine-sensitive and chloroquine-resistant *Plasmodium falciparum* strains (Jonckers et al., 2002; Van et al., 2004). Interestingly, for all compounds, the chloroquine-resistant strain were more sensitive than the chloroquine-sensitive strain. From the results, the main SARs could be summarized (Figure 19): 1) C-1 substitution led to a loss of anti-plasmodial activity; 2) Many of the 2-substituted derivatives displayed higher activity against plasmodia than that of **5** itself but were also more cytotoxic (e.g., **110**). However, the 2-halo-substituted derivatives were more active against *P. falciparum* than that of **5** and less cytotoxic (e.g., **111**); 3) C-3 substituted derivatives possessed about the same or more anti-plasmodial activity against chloroquine-resistant strain, but they were more cytotoxic; 4) C-9 cyano substitution led to a reduction of cytotoxicity but also a loss of the anti-plasmodial activity. Although some **5** derivatives displayed a higher anti-plasmodial activity than **111**, these compounds also showed a more pronounced cytotoxicity. Therefore, compound **111** was considered as the most promising lead for anti-malarial agents in this regard. The mechanism research showed that **111** displayed a low affinity for DNA and no inhibition of human Top II, and this explained the reason for its low cytotoxicity. Further research illustrated this selective anti-plasmodial activity may be associated with the inhibition of β-hematin formation.

**FIGURE 20:**
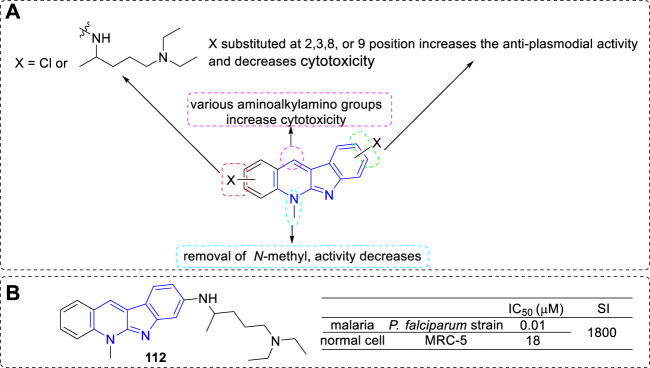
**(A)** SARs of **5** derivatives with basic (aminoalkylamino) side chains; **(B)** Structure and bioactivity of the representative compound **112**.

On further exploration of the anti-malarial potential of **5** derivatives, they introduced halo-substituents as in **111** to reduce the cytotoxicity of the parent compound. In addition, they introduced basic (aminoalkylamino) side chains with the aim to improve the biological activity, as a basic side chain was required for the accumulation of components into the food vacuole, and required for inhibition of hemozoin formation, which was an important character for the activity of chloroquine (El et al., 2009). Hence, a series of **5** derivatives with an *N* ^1^ *,N* ^1^-diethylpentane-1,4-diamine chain (the basic side chain of chloroquine), or other aminoalkylamino chains, chloro-substituents, and a combination of both in various positions were prepared. Then, all the analogues were evaluated for their anti-plasmodial activity against a chloroquine-sensitive *P. falciparum* strain and for cytotoxicity against MRC5 cell line. Most of the compounds showed anti-plasmodial activity in the nanomolar range. According to the activity data, the main SARs were summarized (Figure 20): 1) Most halo-substituted analogs were indeed less cytotoxic than their parent compounds, but also showed lower anti-plasmodial activity; 2) Substituted with the basic *N* ^1^ *,N* ^1^-diethylpentane-1,4-diamine side chain led to a substantial increase of the anti-plasmodial activity, and the compound with basic chain substituted at C-8 position (**112**) appeared the most potent; 3) Compounds removal of the 5-methyl group only resulted in a 1–3 fold loss in potency (still potency), which indicated not the *N*-methyl moiety, but rather the existence of a basic nitrogen atom, was critical for biological activity; 4) Compounds substituted other various aminoalkylaminogroups at C-11 position led to a remarkable increase in cytotoxicity.

**FIGURE 21:**
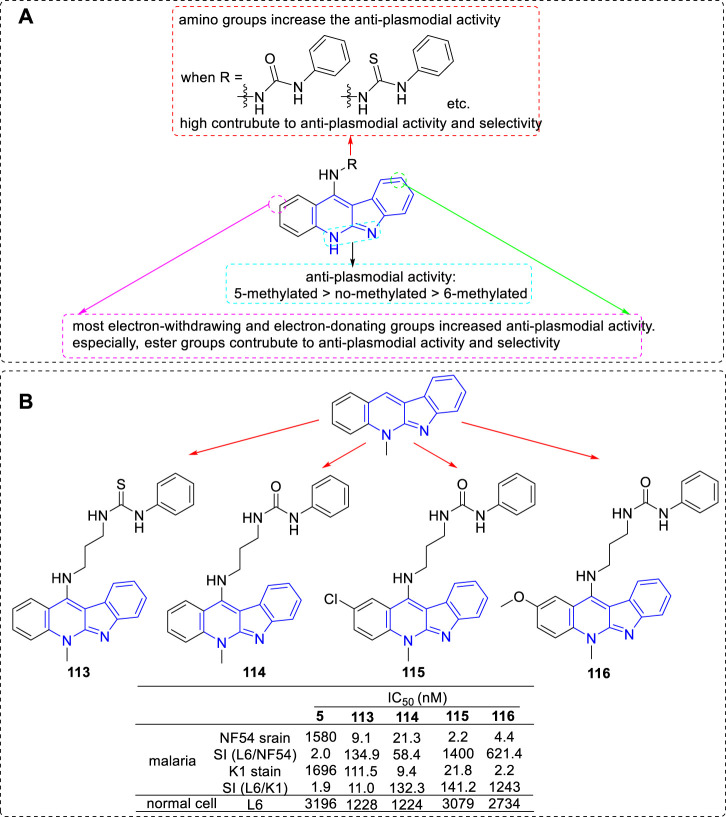
**(A)** SARs of **5** derivatives with various functional groups at C-11 position; **(B)** Structures and bioactivity of the representative compounds **113**–**116**.

Another research group, the team of Inokuchi, has also been committed to modifying **5** in order to improve its anti-plasmodial activity. In 2012, they obtained a series of derivatives by introducing various functional groups at C-11 position (Mei et al., 2013). These functional groups included thiazolidin-4-one (a biologically privileged skeleton which is well tolerated in human subjects), sulfonamide, thiophene-2-carboxamide, and urea/thiourea (aim to improve the solubility properties and the anti-parasitic activity *in vitro*). All of the derivatives were tested for their anti-plasmodial activities toward CQS (NF54) and CQR (K1) of *Plasmodium falciparum* and for cytotoxicity against mammalian L6 cells. The results revealed that urea derivatives highly contributed to anti-plasmodial activity and selectivity. In 2013, the research group continually carried out modifications by fixing a urea/thiourea unit at C-11 position and introducing ester groups at the C2 and/or C9 positions on the core structure of **5** (Lu et al., 2013; Wang et al., 2013). The results illustrated that the ester substituted derivatives not only possessed higher anti-plasmodial activity against both strains, but also a low cytotoxic activity against L6 cells. In the same year, they reported a set of 6-methyl-5*H*-indolo[2,3-*b*]quinoline (congener of **5**) derivatives. These derivatives also were substituted with various alkylamino or *ω*-aminoalkylamino groups at C-11 position. The results suggested that the activity of 6-methylated derivatives were less potent than that of the corresponding 5-methylated derivatives. Based on the sequential reports, the main SARs could be summarized (Figure 21A): 1) The introduction of an amino group at the C-11 position could significantly increase the anti-plasmodial activity compared with the nonsubstituted analogs; 2) Protected nonbasic nitrogen at the terminal of amino group substantially affected the anti-plasmodial activity. These derivatives with nonbasic groups possessed higher select index (SI) data compared with that of the derivatives with free terminal amine substituents. Especially, a urea/thiourea unit highly contributed to anti-plasmodial activity and selectivity; 3) Both electron-withdrawing and electron-donating groups introduced at C-2 and/or C-9 positions increased anti-plasmodial activity. But some of them also increased cytoxicity. Especially, ester groups were favourable for anti-plasmodial activity and selectivity; 4) The anti-plasmodial activity with or without N-methyl group was assigned in the order of 5-methylated > no-methylated > 6-methylated. Several representative compounds (**113–116**) and their activity are shown in Figure 21B. Among these compounds, **113–115** were selected for further study against *Plasmodium berghei* in Swiss mice. After intraperitoneal for four consecutive days at the dose of 50 mg/kg, compounds **113** and **114** showed some reduction of 15.4 and 22.1% in parasitaemia on day 4, respectively. Unfortunately, compound **115** showed no activity and all mice lost weight.

In the discovery of novel compounds containing quinoline core fused five-membered ring structures as anti-tumor and anti-plasmodial agents, Akkachairin *et al.* inadvertently found a novel derivative of compound **5** (**117**) with moderate anti-plasmodial activity but high selectivity (Akkachairin et al., 2020) (Supplementary Figure S5).

#### 4.2.2 Anti-bacterial activity

The team of Kaczmarek not only explored the anti-tumor activity but also the anti-microbial activity of α-carbolines. Early in 1986, they reported certain iso-α-carbolines (e.g., **118**) exhibited anti-bacterial (Gram-positive *Micrococcus luteus* and *Kitasatossporia setae* strains) in the concentration of 0.5 μM/ml (Czoch et al., 1986) (Supplementary Figure S6).

Other α-carbolines derivatives with anti-bacterial activity were seen in Section 4.1.1 (Figures 5, 6, 9).

#### 4.2.3 Anti-fungal activity

Certain α-carbolines derivatives with anti-fungal activity were seen in Section 4.1.1 and Section 4.2.2 (Figures 5, 6, 9, 10; Supplementary Figure S6).

**FIGURE 22:**
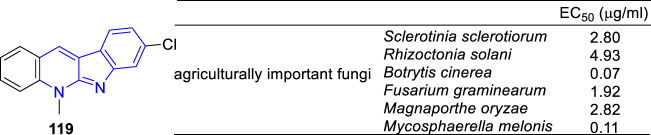
Structure and anti-fungal activity of the representative compound **119**.

Besides the anti-pathogenic fungi of α-carbolines derivatives mentioned above, the anti-agriculturally fungi activity was also reported by Zhu *et.al* (Zhu et al., 2020). They designed and synthesized a series of **5** derivatives and screened their anti-fungal activity against six agriculturally important fungi, including *Rhizoctonia solani*, *Botrytis cinerea* (*B. cinerea*), *Fusarium graminearum*, *Mycosphaerella melonis*, *Sclerotinia sclerotiorum*, and *Magnaporthe oryzae*. Many of these analogs presented remarkable anti-fungal activity with EC_50_ values lower than 1 μg/ml. Notably, compound **119** exhibited the most effective inhibitory potency against *B. cinerea* with an EC_50_ value of 0.07 μg/ml (Figure 22). Subsequently, they carried out its mechanism research through integrating proteomics and transcriptomics (Shang et al., 2021). And the results indicated that it caused the death of *R. solani mycelia* by binding UQCRFS1 and blocking the ion transfer.

#### 4.2.4 Anti-trypanosomal activity

Some indolo[2,3-*b*]quinoline analogs prepared by the research team of Pieters also exhibited anti-trypanosomal activity against *T. cruzi* and *Trypanosoma brucei* in the micromolar range and no obvious cytotoxicity was observed (Jonckers et al., 2002) (Section 4.2.1; Figure 19).

### 4.3 Anti-alzheimer’s disease activity

Alzheimer’s disease (AD) is a progressive neurodegenerative disease resulting in memory loss, disorientation, speech failure and behavioral changes (Selkoe, 2001; Querfurth and LaFerla, 2010), leading to a significant burden to public health systems worldwide (Bosboom et al., 2012). Amyloid β-protein (Aβ) as well as acetylcholinesterase (AChE) and butyrylcholinesterase (BuChE) enzymes are associated with pathology of AD (Adlard et al., 2009; Craig et al., 2011).

**FIGURE 23:**
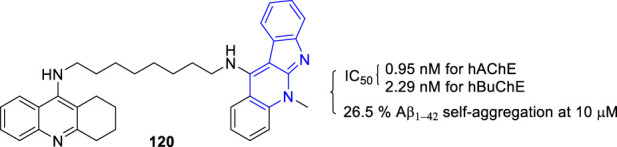
Structure and activity of the representative compound **120**.

The team of Inokuchi synthesized novel tacrine–**5** heterodimers and evaluated their activity towards Aβ as well as AChE and BuChE (Wang et al., 2017). The most potent compound **120** showing a moderate inhibition of the Aβ_1–42_ self-aggregation (26.5% at 10 μM), and a high inhibition of AChE and BuChE with IC_50_ values of 0.95 and 2.29 nM, respectively (Figure 23).

### 4.4 Anti-atherosclerosis activity

Implitapide (**6**), a microsomal triglyceride transfer protein (MTP) inhibitor, had been shown to reduce progression of atherosclerosis (MartinL et al., 2000; Ueshima et al., 2005). At the dose of 12 mg/kg to low-density lipoprotein (LDL)-receptor-deficient Watanabe heritable hyperlipidemic (WHHL) rabbits, the plasma cholesterol level, triglyceride level, and the very low-density lipoprotein VLDL secretion rate were decreased by 70, 45, and 80%, respectively.

### 4.5 Antioxidant activity

**FIGURE 24:**
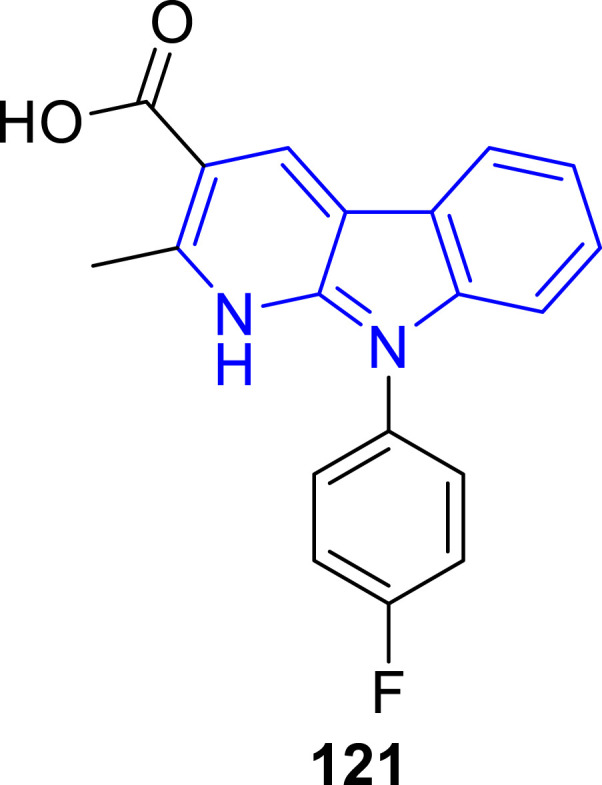
Structure of the representative compound **121**.

Recently, Zhang and coworkers (Zhang et al., 2022) designed and synthesized a series of α-carboline derivatives to improve the damage of cardiomyocyte caused by oxidative stress. The biological studies showed that most of the α-carbolines exhibited obvious protective activities against H_2_O_2_-induced cardiomyocyte injury. Particularly, compound **121** signifcantly increased the cell viability in H_2_O_2_-induced oxidative stress in H9c2 cardiomyoblasts with a concentration-dependent manner. Other biological results including measurement of the activities of MDA, SOD, and GSH-Px, flow cytometry analysis, and Western blot analysis also revealed the potential of **121** as a promising cardioprotective agent against H_2_O_2_-induced oxidative injury (Figure 24).

Therefore, these analogs may be used to treat many oxidation related diseases such as cancer, cardiovascular, and inflammation caused by oxidative stress (Altwaijry et al., 2021).

### 4.6 Miscellaneous

Besides the activities mentioned above, α-carboline derivatives also showed other biological activities. Amino-α-carboline, which was formed during the cooking of meat or fish, could be used as mutagens due to its genotoxicity (Yoshida et al., 1979; Zhang et al., 1996). Mescengricin (**9**), was first found in the process of screening for neuronal cell protecting components, showed protection of chick primary mesencephalic neurons from L-glutamatc toxicity, suggesting this class of α-carbolines possess a potential of neuroprotective activity (Shin et al., 2000). DDMG-1 (**10**) could inhibit the mRNA degradation of IκB-α, mTNF-α, and influence other inflammation related factors including NF-κB, IL-8, etc., indicating this class of anologs should be further researched for the treatment of chronic inflammatory diseases (Oda et al., 2009). Some α-carbolines were used to develop organic semiconductors (Han et al., 2015) and host materials (Hwang et al., 2020) due to their highly planar, rigid, polycyclic chromophore structure and sufficiently large triplet energy.

## 5 Conclusion and future directions

**FIGURE 25:**
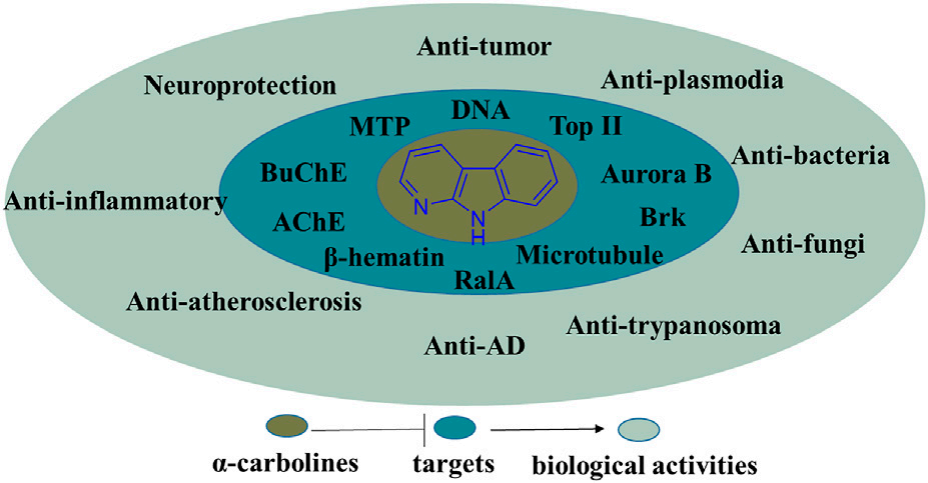
Summary of the biological activities and their targets possessed by α-carboline derivatives.

In summary, the α-carboline natural products, recent literature of synthesis and information of the biological activities possessed by α-carboline alkaloids have been presented in the review. In the section of biological activities, some SARs and activity mechanisms had been described. Undoubtedly, α-carboline derivatives exert diverse biological activities, suggesting this class of alkaloids have great potential in medicinal chemistry (Figure 25). Although there are no α-carboline-based drugs on the market currently, some α-carboline-based compounds have reached clinical trials (implitapide, **85** for example). The research of α-carbolines as drugs should be going.

Still, there will be a long way to go before α-cabolines become drugs, and incontrovertibly many challenges will be faced. The first challenge is their synthesis. Although a broad array of strategies have been reported for their synthesis, it is surprising that few literature reported the total synthesis of a-carboline natural products. Many of the synthetic methods of a-carbolines described are limited by low yields, starting materials difficult to obtain, expensive reagents, or inflexibility with regard to substituents and substitution patterns. The second challenge is their activity. It can be found from literature that many novel α-cabolines have been synthesized, but their activity is poor or mediocre. The third challenge is their clinical research. Although some teams have studied α-cabolines for decades, and some compounds possessed good activity, there are still no compounds reached clinical trials.

Neverthless, some future directions of α-carboline-based medicinal chemistry could be summarized as follows: 1) Efficient and facile synthesis methods still need to develop; 2) The structural modification of active α-carbolines still needs to be carried out. On the one hand, to improve their physicochemical properties or selectivity, on the other hand, to broaden the scope of compounds for diverse biological activity; 3) Recent biological activity research of α-carbolines mainly focuses on anti-tumor activity, research on other biological activities should be reinforced; 4) The mechanisms of their biological activities should be elucidated, especially their target should be clear; 5) Due to their polycyclic chromophore structure, making α-carbolines into medical materials is also an important direction.

## Abbreviations

Aβ, amyloid β-protein; AchE, acetylcholinesterase; AD, Alzheimer’s disease; ALK, Anaplastic Lymphoma Kinase; BuChE, butyrylcholinesterase; ACTs, artemisinin-based combination therapies; Brk, breast tumor kinase; DiMIQ, 5,11-dimethy-5H-indolo[2,3-b]quinoline; DPPH, 2,2-Diphenyl-1-picrylhydrazyl; ED50, 50% effective dose; IC50, 50% in hibition concentration; ID50, 50% infective dose; LDL, low-density lipoprotein; MDR, Multidrug resisitance; MIC, Minimum inhibitory concentration; MTP, microsomal triglyceride transfer protein; PARP, poly ADP ribose polymerase; RalA, ras-related protein; SAR, structure activity relationship; SI, select index; Top, topoisomerase; VLDL, very low-density lipoprotein; WHHL, Watanabe heritable hyperlipidemic.

## References

[B1] Adlard P. James S. Bush A. Masters C. (2009). beta-Amyloid as a molecular therapeutic target in Alzheimer's disease. Drugs Today (Barc) 45, 293–304. 10.1358/dot.2009.45.4.1353853 19499094

[B2] Akkachairin B. Rodphon W. Reamtong O. Mungthin M. Tummatorn J. Thongsornkleeb C. (2020). Synthesis of neocryptolepines and carbocycle-fused quinolines and evaluation of their anticancer and antiplasmodial activities. Bioorg. Chem. 98, 103732. 10.1016/j.bioorg.2020.103732 32171989

[B3] Altwaijry N. El-Ghlban S. El Sayed I. E. El-Bahnsawye M. Bayomi A. I. Samaka R. M. (2021). *In vitro* and *in vivo* antitumor activity of indolo[2, 3-b] quinolines, natural product analogs from neocryptolepine alkaloid. Molecules 26, 754–775. 10.3390/molecules26030754 33535575 PMC7867085

[B4] Barker K. Jackson L. Crompton M. (1997). BRK tyrosine kinase expression in a high proportion of human breast carcinomas. Oncogene 15, 799–805. 10.1038/sj.onc.1201241 9266966

[B5] Bednarek E. Bocian W. Sitkowski J. Ulkowska A. Kaczmarek L. Badowska-Roslonek K. (2006). 1H and 13C NMR data for indolo[2, 3-b]quinoline-aminoglycoside hybrids, a novel potent anticancer drug family. Magn. Reson. Chem. 44, 459–462. 10.1002/mrc.1737 16425213

[B6] Bosboom P. Alfonso H. Eaton J. Almeida O. (2012). Quality of life in Alzheimer's disease: Different factors associated with complementary ratings by patients and family carers. Int. Psychogeriatr. 24, 708–721. 10.1017/S1041610211002493 22244307

[B7] Brauer P. Tyner A. (2010). Building a better understanding of the intracellular tyrosine kinase PTK6 - BRK by BRK. Biochimica Biophysica Acta - Rev. Cancer 1806, 66–73. 10.1016/j.bbcan.2010.02.003 PMC2885473 20193745

[B8] Bum-Erdene K. Liu D. Gonzalez-Gutierrez G. Ghozayel M. Xu D. Meroueh S. (2020). Small-molecule covalent bond formation at tyrosine creates a binding site and inhibits activation of Ral GTPases. Proc. Natl. Acad. Sci. U. S. A. 117, 7131–7139. 10.1073/pnas.1913654117 32179690 PMC7132301

[B9] Carmena M. Earnshaw W. (2003). The cellular geography of aurora kinases. Nat. Rev. Mol. Cell Biol. 4, 842–854. 10.1038/nrm1245 14625535

[B10] Carmena M. Ruchaud S. Earnshaw W. (2009). Making the auroras glow: Regulation of aurora A and B kinase function by interacting proteins. Curr. Opin. Cell Biol. 21, 796–805. 10.1016/j.ceb.2009.09.008 19836940 PMC2806521

[B11] Chen F. Li X. Zhu H. Huang W. (2020). Regulation of the ras-related signaling pathway by small molecules containing an indole core scaffold: A potential antitumor therapy. Front. Pharmacol. 11, 280. 10.3389/fphar.2020.00280 32231571 PMC7082308

[B12] Chen S. Chan N. Hsieh T. (2013). New mechanistic and functional insights into DNA topoisomerases. Annu. Rev. Biochem. 82, 139–170. 10.1146/annurev-biochem-061809-100002 23495937

[B13] Cimanga K. Bruyne T. D. Pieters L. Claeys M. Vlietinck A. (1996). New alkaloids from Cryptolepis sanguinolenta. Tetrahedron Lett. 37, 1703–1706. 10.1016/0040-4039(96)00112-8

[B14] Cimanga K. Bruyne T. D. Pieters L. Vlietinck A. J. Turger C. A. (1997). *In vitro* and *in vivo* antiplasmodial activity of cryptolepine and related alkaloids from cryptolepis sanguinolenta. J. Nat. Prod. 60, 688–691. 10.1021/np9605246 9249972

[B15] Cimanga K. De Bruyne T. Pieters L. Totte J. Tona L. Kambu K. (1998). Antibacterial and antifungal activities of neocryptolepine, biscryptolepine and cryptoquindoline, alkaloids isolated from Cryptolepis sanguinolenta. Phytomedicine 5, 209–214. 10.1016/S0944-7113(98)80030-5 23195843

[B16] Craig L. Hong N. McDonald R. (2011). Revisiting the cholinergic hypothesis in the development of Alzheimer's disease. Neurosci. Biobehav. Rev. 35, 1397–1409. 10.1016/j.neubiorev.2011.03.001 21392524

[B17] Czoch W. P. Mordarski M. Kaczmarek Ł. Namirski P. N. (1986). Structure-activity relationship studies on selected iso-alpha-carbolines. Arch. Immunol. Ther. Exp. 34, 327–331. 3296992

[B18] Czoch W. P. Pognan F. Kaczmarek Ł. Boratyñski J. (1994). Synthesis and structure -activity relationship of methyl-substituted indolo[2, 3-b]quinolines:Novel cytotoxic, DNA topoisomerase II inhibitors. J. Med. Chem. 37, 3503–3510. 10.1021/jm00047a008 7932579

[B19] Dai J. Dan W. Zhang Y. Wang J. (2018). Recent developments on synthesis and biological activities of gamma-carboline. Eur. J. Med. Chem. 157, 447–461. 10.1016/j.ejmech.2018.08.015 30103193

[B20] Debnath S. Kumar A. S. Chauhan S. Kumara Swamy K. C. (2021). Divergent reactivity of delta- and beta'-acetoxy allenoates with 2-sulfonamidoindoles via phosphine catalysis: Entry to dihydro-alpha-carboline, alpha-carboline, and spiro-cyclopentene motifs. J. Org. Chem. 86, 11583–11598. 10.1021/acs.joc.1c01137 34343010

[B21] Efferth T. (2006). Molecular pharmacology and pharmacogenomics of artemisinin and its derivatives in cancer cells. Curr. drug targets 7, 407–421. 10.2174/138945006776359412 16611029

[B22] El S. I. Van der Veken P. Steert K. Dhooghe L. Hostyn S. Van Baelen G. (2009). Synthesis and antiplasmodial activity of aminoalkylamino-substituted neocryptolepine derivatives. J. Med. Chem. 52, 2979–2988. 10.1021/jm801490z 19364118

[B23] Emam S. M. El Sayed Iel T. Nassar N. (2015). Transition metal complexes of neocryptolepine analogues. Part I: Synthesis, spectroscopic characterization, and invitro anticancer activity of copper(II) complexes. Spectrochimica Acta Part A Mol. Biomol. Spectrosc. 138, 942–953. 10.1016/j.saa.2014.03.114 24867072

[B24] Fan G. Lou L. Song Z. Zhang X. Xiong X. (2021). Targeting mutated GTPase KRAS in tumor therapies. Eur. J. Med. Chem. 226, 113816. 10.1016/j.ejmech.2021.113816 34520956

[B25] Farrell P. Shi L. Matuszkiewicz J. Balakrishna D. Hoshino T. Zhang L. (2013). Biological characterization of TAK-901, an investigational, novel, multitargeted Aurora B kinase inhibitor. Mol. Cancer Ther. 12, 460–470. 10.1158/1535-7163.MCT-12-0657 23358665

[B26] Fu J. Bian M. Jiang Q. Zhang C. (2007). Roles of Aurora kinases in mitosis and tumorigenesis. Mol. cancer Res. 5, 1–10. 10.1158/1541-7786.MCR-06-0208 17259342

[B27] Godlewska J. Luniewski W. Zagrodzki B. Kaczmarek Ł. Pohl A. B. Dus D. (2005). Biological evaluation of omega-(dialkylamino)alkyl derivatives of 6H-indolo[2, 3-b]quinoline--novel cytotoxic DNA topoisomerase II inhibitors. Anticancer Res. 25, 2857–2868. 16080538

[B28] Goto Y. Kamihira R. Nakao Y. Nonaka M. Takano R. Xuan X. (2021). The efficacy of marine natural products against Plasmodium falciparum. J. Parasitol. 107, 284–288. 10.1645/20-93 33844839

[B29] Han J. Thirupathaiah B. Kwon G. Kim C. Seo S. (2015). Synthesis and characterization of carbazole- and α-carboline-based thiophene derivatives as organic semiconductors for organic thin-film transistors. Dyes Pigm. 114, 78–84. 10.1016/j.dyepig.2014.10.024

[B30] Huang H. C. Liu W. T. Hua K. S. Hung H. C. Tsai J. Y. Kuo S. C. (2016). α-Carboline derivative TJY-16 inhibits tumor growth by inducing G2/M cell cycle arrest in glioma cells. J. Biomed. Sci. 23, 10–21. 10.1186/s12929-016-0222-y 26786523 PMC4717554

[B31] Humeniuk R. Kaczmarek Ł. Peczyñska-Czoch W. Marcinkowska E. (2002). Cytotoxicity and cell cycle effects of novel indolo[2, 3-** *b* **]quinoline derivatives. Oncol. Res. 13, 269–277. 10.3727/096504003108748320 12688678

[B32] Hunter J. Manandhar A. Carrasco M. Gurbani D. Gondi S. Westover K. (2015). Biochemical and structural analysis of common cancer-associated KRAS mutations. Mol. Cancer Res. 13, 1325–1335. 10.1158/1541-7786.MCR-15-0203 26037647

[B33] Hwang J. Lee C. Jeong J. E. Kim C. Y. Woo H. Y. Park S. (2020). Rational design of carbazole- and carboline-based polymeric host materials for realizing high-efficiency solution-processed thermally activated delayed fluorescence organic light-emitting diode. ACS Appl. Mat. Interfaces 12, 8485–8494. 10.1021/acsami.9b20279 31990169

[B34] Jackson J. Patrick D. Dar M. Huang P. (2007). Targeted anti-mitotic therapies: Can we improve on tubulin agents? Nat. Rev. Cancer 7, 107–117. 10.1038/nrc2049 17251917

[B35] Jonckers T. H. M. Miert S. V. Cimanga K. Bailly C. Colson P. Pauw-Gillet M. C. D. (2002). Synthesis, cytotoxicity, and antiplasmodial and antitrypanosomal activity of new neocryptolepine derivatives. J. Med. Chem. 45, 3497–3508. 10.1021/jm011102i 12139461

[B36] Kaczmarek Ł. Balicki R. Namirski P. N. Peczynska-Czoch W. MordarskiM. (1988a). Cancerostatics, VI. Synthesis and antineoplastic properties of some benzo-iso-alpha-carbolines. Arch. Pharm. 321, 463–467. 10.1002/ardp.19883210807 3223804

[B37] Kaczmarek Ł. Czoch W. P. Opolski A. Wietrzyk J. Marciniowska E. Boratyñski J. (1998b). Methoxy- and methyl-methoxy-5, 6, 11-trimethyl-6H-indolo [2, 3-b]quinolinium derivatives as novel cytotoxic agents and DNA topoisomerase II inhibitors. Anticancer Res. 18, 3133–3138. 9713522

[B38] Kaczmarek Ł. Czoch W. P. Osiadacz J. Mordarski M. Sokalski W. A. Boratyñski J. (1999). Synthesis, and cytotoxic activity of some novel indolo[2, 3-b]quinoline derivatives DNA topoisomerase II inhibitors. Bioorg. Med. Chem. 7, 2457–2464. 10.1016/s0968-0896(99)00200-x 10632055

[B39] Kaczmarek Ł. Łuniewski W. Zagrodzki B. Godlewska J. Osiadacz J. Wietrzyk J. (2002). Synthesis of 6-substituted 6H-indolo[2, 3-b]quinolines as novel cytotoxic agents and topoisomerase II inhibitors. Acta Pol. Pharm. 59, 199–207. 12230247

[B40] Kamihira R. Nakao Y. (2021). Small-scale preparation of fluorescently labeled chemical probes from marine cyclic peptides, kapakahines A and F. Mar. Drugs 19, 76–87. 10.3390/md19020076 33572527 PMC7912572

[B41] Kaur R. Kaur G. Gill R. Soni R. Bariwal J. (2014). Recent developments in tubulin polymerization inhibitors: An overview. Eur. J. Med. Chem. 87, 89–124. 10.1016/j.ejmech.2014.09.051 25240869

[B42] Kavallaris M. (2010). Microtubules and resistance to tubulin-binding agents. Nat. Rev. Cancer 10, 194–204. 10.1038/nrc2803 20147901

[B43] Kim J.-S. Shin-ya K. Hayakawa Y. Seto H. (1997). Structure of mescengricin, A novel neuronal cell protecting substance produced by streptorayces griseoflavus. Tetrahedron Lett. 38, 3431–3434. 10.1080/10286020008039901

[B44] Lavrado J. Moreira R. Paulo A. (2010). Indoloquinolines as scaffolds for drug discovery. Curr. Med. Chem. 17, 2348–2370. 10.2174/092986710791698521 20491639

[B45] Leng H. J. Wang Y. T. He X. H. Xia H. L. Xu P. S. Xiang P. (2020). Design and efficient synthesis of RalA inhibitors containing the dihydro-alpha-carboline scaffold. ChemMedChem 16, 851–859. 10.1002/cmdc.202000722 33244883

[B46] Li D. Liu W. Huang Y. Liu M. Tian C. Lu H. (2022). Facile synthesis of C1-substituted beta-carbolines as CDK4 inhibitors for the treatment of cancer. Bioorg. Chem. 121, 105659. 10.1016/j.bioorg.2022.105659 35180487

[B47] Liang X. Wu Q. Luan S. Yin Z. He C. Yin L. (2019). A comprehensive review of topoisomerase inhibitors as anticancer agents in the past decade. Eur. J. Med. Chem. 171, 129–168. 10.1016/j.ejmech.2019.03.034 30917303

[B48] Lin Y. C. Chen Y. F. Tseng L. S. Lee Y. H. Morris-Natschke S. L. Kuo S. C. (2016). Synthesis and structure-activity relationship studies of novel 3, 9-substituted alpha-carboline derivatives with high cytotoxic activity against colorectal cancer cells. Eur. J. Med. Chem. 110, 98–114. 10.1016/j.ejmech.2016.01.004 26820553 PMC4754133

[B49] Liu W. Liu X. Liu W. Gao Y. Wu L. Huang Y. (2022). Discovery of novel β-carboline derivatives as selective AChE inhibitors with GSK-3β inhibitory property for the treatment of Alzheimer's disease. Eur. J. Med. Chem. 229, 114095. 10.1016/j.ejmech.2021.114095 34995924

[B50] Liu W. Liu X. Tian L. Gao Y. Liu W. Chen H. (2021). Design, synthesis and biological evaluation of harmine derivatives as potent GSK-3β/DYRK1A dual inhibitors for the treatment of Alzheimer's disease. Eur. J. Med. Chem. 222, 113554. 10.1016/j.ejmech.2021.113554 34098466

[B51] Lu W. J. Wicht K. J. Wang L. Imai K. Mei Z. W. Kaiser M. (2013). Synthesis and antimalarial testing of neocryptolepine analogues: Addition of ester function in SAR study of 2, 11-disubstituted indolo[2, 3-b]quinolines. Eur. J. Med. Chem. 64, 498–511. 10.1016/j.ejmech.2013.03.072 23685569

[B52] Luniewski W. Wietrzyk J. Godlewska J. Switalska M. Piskozub M. Peczynska-Czoch W. (2012). New derivatives of 11-methyl-6-[2-(dimethylamino)ethyl]-6H-indolo[2, 3-b]quinoline as cytotoxic DNA topoisomerase II inhibitors. Bioorg. Med. Chem. Lett. 22, 6103–6107. 10.1016/j.bmcl.2012.08.032 22944121

[B53] Mahmoud K. A. Krug M. Wersig T. Slynko I. Schachtele C. Totzke F. (2014). Discovery of 4-anilino alpha-carbolines as novel Brk inhibitors. Bioorg. Med. Chem. Lett. 24, 1948–1951. 10.1016/j.bmcl.2014.03.002 24650640

[B54] MartinL L. Sorbera A. Silvestre J. Castañer J. (2000). Implitapide. Hypolipidemic, Treatment of atherosclerosis, MTP inhibitor, ApoB secretion inhibitor. Drugs Future 25, 1138–1144.

[B55] Medas K. M. Lesch R. W. Edioma F. B. Wrenn S. P. Ndahayo V. Mulcahy S. P. (2020). Metal-catalyzed cyclotrimerization reactions of cyanamides: Synthesis of 2-Aryl-alpha-carbolines. Org. Lett. 22, 3135–3139. 10.1021/acs.orglett.0c00891 32255636 PMC7895322

[B56] Mei Z. W. Wang L. Lu W. J. Pang C. Q. Maeda T. Peng W. (2013). Synthesis and *in vitro* antimalarial testing of neocryptolepines: SAR study for improved activity by introduction and modifications of side chains at C2 and C11 on indolo[2, 3-b]quinolines. J. Med. Chem. 56, 1431–1442. 10.1021/jm300887b 23360309

[B57] Mineno M. Sera M. Ueda T. Mizufune H. Zanka A. O'Bryan C. (2015). Integrated cross-coupling strategy for an alpha-carboline-based Aurora B kinase inhibitor. J. Org. Chem. 80, 1564–1568. 10.1021/jo502489x 25616084

[B58] Mitchell P. Barker K. Martindale J. Kamalati T. Lowe P. Page M. (1994). Cloning and characterisation of cDNAs encoding a novel non-receptor tyrosine kinase, brk, expressed in human breast tumours. Oncogene 9, 2383–2390. 8036022

[B59] Mologni L. Orsato A. Zambon A. Tardy S. Bisson W. H. Schneider C. (2022a). Identification of non-ATP-competitive α-carboline inhibitors of the anaplastic lymphoma kinase. Eur. J. Med. Chem. 238, 114488. 10.1016/j.ejmech.2022.114488 35665691

[B60] Mologni L. Tardy S. Zambon A. Orsato A. Bisson W. Ceccon M. (2022b). Discovery of novel α-carboline inhibitors of the anaplastic lymphoma kinase. ACS Omega 7, 17083–17097. 10.1021/acsomega.2c00507 35647450 PMC9134258

[B61] Morrissey C. Gallis B. Solazzi J. Kim B. Gulati R. Vakar-Lopez F. (2010). Effect of artemisinin derivatives on apoptosis and cell cycle in prostate cancer cells. Anticancer. Drugs 21, 423–432. 10.1097/CAD.0b013e328336f57b 20130467 PMC2953769

[B62] Nakao Y. Kuo J. Yoshida W. Y. Kelly M. Scheuer P. J. (2003). More kapakahines from the marine sponge Cribrochalina olemda. Org. Lett. 5, 1387–1390. 10.1021/ol026830u 12713280

[B63] Nantka N. P. Kaczmarek Ł. (1978). Synthesis and preliminary cytostatic screening of some alpha-carboline derivatives. Pol. J. Pharmacol. Pharm. 30, 569–572. 740558

[B64] Nitiss J. (2009). Targeting DNA topoisomerase II in cancer chemotherapy. Nat. Rev. Cancer 9, 338–350. 10.1038/nrc2607 19377506 PMC2748742

[B65] Nuthakki V. K. Mudududdla R. Bharate S. B. (2022). Role of basic aminoalkyl chains in the lead optimization of Indoloquinoline alkaloids. Eur. J. Med. Chem. 227, 113938. 10.1016/j.ejmech.2021.113938 34710743

[B66] Oda T. Lee J. S. Sato Y. Kabe Y. Sakamoto S. Handa H. (2009). Inhibitory effect of N, N-didesmethylgrossularine-1 on inflammatory cytokine production in lipopolysaccharide-stimulated RAW 264.7 cells. Mar. Drugs 7, 589–599. 10.3390/md7040589 20098600 PMC2810236

[B67] Oelze M. Mahmoud K. A. Sippl W. Wersig T. Hilgeroth A. Ritter C. A. (2015). Novel 4-anilino-alpha-carboline derivatives induce cell death in nonadhesive breast cancer cells through inhibition of Brk activity. Int. J. Clin. Pharmacol. Ther. 53, 1052–1055. 10.5414/CPXCES14EA07 26396134

[B68] Pattey C. M. Guyot M. (1989). Grossularine-1 and grossularine-2, cytotoxic α-carbolines from the tunicate: Dendrodoa grossularia. Tetrahedron 45, 3445–3450. 10.1016/s0040-4020(01)81023-1

[B69] Pognan F. Saucier J.-M. Paoletti C. Kaczmarek L. Namirski P. N. Mordarski M. (1992). A carboline derivative as a novel mammalian DNA topoisomerase II targeting agent. Biochem. Pharmacol. 44, 2149–2155. 10.1016/0006-2952(92)90341-f 1335251

[B70] Pradhan T. Gupta O. Singh G. Monga V. (2021). Aurora kinase inhibitors as potential anticancer agents: Recent advances. Eur. J. Med. Chem. 221, 113495. 10.1016/j.ejmech.2021.113495 34020340

[B71] Querfurth H. W. LaFerla F. M. (2010). Alzheimer's disease. N. Engl. J. Med. 362, 329–344. 10.1056/nejmra0909142 20107219

[B72] Rocha D. D. Espejo V. R. Rainier J. D. La Clair J. J. Costa-Lotufo L. V. (2015). Fluorescent kapakahines serve as non-toxic probes for live cell Golgi imaging. Life Sci. 136, 163–167. 10.1016/j.lfs.2015.06.014 26141988

[B73] Roslonek K. B. Ciesielska A. Šwitalska M. Piskozub M. P.-Czoch W. Wietrzyk J. (2016). Synthises and cytotoxic activity of new 5H-indolo[2, 3-b]quinoline O-aminoglycosides. Acta Pol. Pharm. 73, 683–692. 27476287

[B74] Selkoe D. (2001). Alzheimer's disease: Genes, proteins, and therapy. Physiol. Rev. 81, 741–766. 10.1152/physrev.2001.81.2.741 11274343

[B75] Shaban E. Switalska M. Wang L. Wang N. Xiu F. Hayashi I. (2017). Synthesis and *in vitro* antiproliferative activity of 11-substituted neocryptolepines with a branched omega-aminoalkylamino chain. Molecules 22, 1954. 10.3390/molecules22111954 PMC6150407 29137152

[B76] Shang X. F. Dai L. X. Zhang Z. J. Yang C. J. Du S. S. Wu T. L. (2021). Integrated proteomics and transcriptomics analyses reveals the possible antifungal mechanism of an indoloquinoline alkaloid neocryptolepine against Rhizoctonia solani. J. Agric. Food Chem. 69, 6455–6464. 10.1021/acs.jafc.1c01385 34075744

[B77] Sharaf H. M. Schiff P. L. Tackie A. N. Phoebe C. H. Martin G. E. (1996). Two new indoloquinoline alkaloids from cryptolepis sanguinolenta: Cryptosanguinolentine and cryptotackieine. J. Heterocycl. Chem. 33, 239–243. 10.1002/jhet.5570330204

[B78] Shin Y. K. Kim J. S. Furihata K. Hayakawa Y. Seto H. (2000). A novel neuronal cell protecting substance mescengricin produced by Streptomyces griseoflavus. J. Asian Nat. Prod. Res. 2, 121–132. 10.1080/10286020008039901 11252677

[B79] Sidoryk K. Jaromin A. Edward J. A. Switalska M. Stefanska J. Cmoch P. (2014). Searching for new derivatives of neocryptolepine: Synthesis, antiproliferative, antimicrobial and antifungal activities. Eur. J. Med. Chem. 78, 304–313. 10.1016/j.ejmech.2014.03.060 24686017

[B80] Sidoryk K. Switalska M. Rozga P. Wietrzyk J. Bujak I. Zerek B. (2017). An efficient synthesis of indolo[2, 3-b]quinoline guanidine derivatives with their *in vitro* and *in vivo* study. Med. Chem. Res. 26, 3354–3366. 10.1007/s00044-017-2028-1 29170613 PMC5676820

[B81] Sidoryk K. Switalska M. Jaromin A. Cmoch P. Bujak I. Kaczmarska M. (2015). The synthesis of indolo[2, 3-b]quinoline derivatives with a guanidine group: Highly selective cytotoxic agents. Eur. J. Med. Chem. 105, 208–219. 10.1016/j.ejmech.2015.10.022 26496013

[B82] Sidoryk K. Switalska M. Wietrzyk J. Jaromin A. Pietka-Ottlik M. Cmoch P. (2012). Synthesis and biological evaluation of new amino acid and dipeptide derivatives of neocryptolepine as anticancer agents. J. Med. Chem. 55, 5077–5087. 10.1021/jm300468t 22574992

[B83] Sung H. Ferlay J. Siegel R. L. Laversanne M. Soerjomataram I. Jemal A. (2021). Global cancer Statistics 2020: GLOBOCAN estimates of incidence and mortality worldwide for 36 cancers in 185 countries. Ca. A Cancer J. Clin. 71, 209–249. 10.3322/caac.21660 33538338

[B84] Tian C. Huang S. Xu Z. Liu W. Li D. Liu M. (2022). Design, synthesis, and biological evaluation of beta-carboline 1, 3, 4-oxadiazole based hybrids as HDAC inhibitors with potential antitumor effects. Bioorg. Med. Chem. Lett. 64, 128663. 10.1016/j.bmcl.2022.128663 35272009

[B85] Tsai J.-Y. Lin Y.-C. Hsu M.-H. Kuo S.-C. Huang L.-J. (2010). Synthesis and cytotoxicity of 1, 6, 8, 9-substituted α-carboline derivatives. Kaohsiung J. Med. Sci. 26, 593–602. 10.1016/s1607-551x(10)70091-7 21126712 PMC11916198

[B86] Tsui T. Miller W. (2015). Cancer-associated mutations in breast tumor kinase/PTK6 differentially affect enzyme activity and substrate recognition. Biochemistry 54, 3173–3182. 10.1021/acs.biochem.5b00303 25940761 PMC4757811

[B87] Ueshima K. Umeno H. A. Nagayoshi K. Takakuar S. Matsuo M. Mutoh S. (2005). Implitapide, a microsomal triglyceride transfer protein inhibitor, reduces progression of atherosclerosis in apolipoprotein E knockout mice fed a western-type diet: Involvement of the inhibition of postprandial triglyceride elevation. Biol. Pharm. Bull. 28, 247–252. 10.1248/bpb.28.247 15684478

[B88] Van M. S. Jonckers T. Cimanga K. Maes L. Maes B. Lemiere G. (2004). *In vitro* inhibition of beta-haematin formation, DNA interactions, antiplasmodial activity, and cytotoxicity of synthetic neocryptolepine derivatives. Exp. Parasitol. 108, 163–168. 10.1016/j.exppara.2004.08.006 15582513

[B89] Verbitski S. M. Mayne C. L. Davis R. A. Concepcion G. P. Ireland C. M. (2002). Isolation, structure determination, and biological activity of a novel alkaloid, perophoramidine, from the philippine ascidian perophoranamei. J. Org. Chem. 67, 7124–7126. 10.1021/jo026012f 12354007

[B90] Wadsworth A. D. Naysmith B. J. Brimble M. A. (2015). A review of the synthesis of alpha-carbolines. Eur. J. Med. Chem. 97, 816–829. 10.1016/j.ejmech.2014.11.038 25499235

[B91] Wang L. Moraleda I. Iriepa I. Romero A. Lopez-Munoz F. Chioua M. (2017). 5-Methyl-N-(8-(5, 6, 7, 8-tetrahydroacridin-9-ylamino)octyl)-5H-indolo[2, 3-b]quinoli n-11-amine: A highly potent human cholinesterase inhibitor. Medchemcomm 8, 1307–1317. 10.1039/c7md00143f 30108842 PMC6071787

[B92] Wang L. Switalska M. Mei Z. W. Lu W. J. Takahara Y. Feng X. W. (2012). Synthesis and *in vitro* antiproliferative activity of new 11-aminoalkylamino-substituted 5H- and 6H-indolo[2, 3-b]quinolines; structure-activity relationships of neocryptolepines and 6-methyl congeners. Bioorg. Med. Chem. 20, 4820–4829. 10.1016/j.bmc.2012.05.054 22748378

[B93] Wang L. Switalska M. Wang N. Du Z. J. Fukumoto Y. Diep N. K. (2014). Design, synthesis, and biological evaluation of artemisinin-indoloquinoline hybrids as potent antiproliferative agents. Molecules 19, 19021–19035. 10.3390/molecules191119021 25412047 PMC6271626

[B94] Wang N. Switalska M. Wang L. Shaban E. Hossain M. I. Sayed I. E. T. E. (2019). Structural modifications of nature-inspired indoloquinolines: A mini review of their potential antiproliferative activity. Molecules 24, 24112121. 10.3390/molecules24112121 PMC6600460 31195640

[B95] Wang N. Wicht K. J. Wang L. Lu W. J. Misumi R. Wang M. Q. (2013). Synthesis and *in vitro* testing of antimalarial activity of non-naturalType neocryptolepines: Structure–activity relationship study of 2, 11and 9, 11-disubstituted 6-methylindolo[2, 3-b]quinolines. Chem. Pharm. Bull. 61, 1282–1290. 10.1248/cpb.c13-00639 24436959

[B96] Wieczorek J. Czoch W. P. Mordarski M. Kaczmarek Ł. Namirski P. N. (1986). Antineoplastic activity of azacarbazoles. I. Synthesis and antitumor properties of alpha-carboline and its selected derivatives. Arch. Immunol. Ther. Exp. 34, 315–321. 3592935

[B97] Yan C. Theodorescu D. (2018). RAL GTPases: Biology and potential as therapeutic targets in cancer. Pharmacol. Rev. 70, 1–11. 10.1124/pr.117.014415 29196555 PMC5712631

[B98] Yan Q. Gin E. Banwell M. G. Willis A. C. Carr P. D. (2017). A unified approach to the isomeric α-β-γ-and δ-carbolines via their 6, 7, 8, 9-tetrahydro counterparts. J. Org. Chem. 82, 4328–4335. 10.1021/acs.joc.7b00323 28304164

[B99] Yeung B. K. Nakao Y. Kinnel R. B. Carney J. R. Yoshida W. Y. Scheuer P. J. (1996). The kapakahines, cyclic peptides from the marine sponge *Cribrochalina olemda* . J. Org. Chem. 61, 7168–7173. 10.1021/jo960725e 11667621

[B100] Yi J. M. Zhang X. F. Huan X. J. Song S. S. Wang W. Tian Q. T. (2015). Dual targeting of microtubule and topoisomerase II by α-carboline derivative YCH337 for tumor proliferation and growth inhibition. Oncotarget 6, 8960–8973. 10.18632/oncotarget.3264 25840421 PMC4496195

[B101] Yoshida D. Matsumoto T. Okamoto H. (1979). Interaction between amino-alpha-carboline and amino-gamma-carboline on mutagenicity in *Salmonella typhimurium* . Mutat. Research/Genetic Toxicol. 68, 175–178. 10.1016/0165-1218(79)90146-0 390389

[B102] Zhang R. H. Yang D. Liao X. M. Zhang H. Chen G. Q. Zhang W. L. (2022). Design, synthesis, and *in vitro* protective effect evaluation of α-carboline derivatives against H2O2-induced cardiomyocyte injury. Eur. J. Med. Chem. 238, 114469. 10.1016/j.ejmech.2022.114469 35605360

[B103] Zhang X. B. Felton J. S. Tucker J. D. Urlando C. Heddle J. A. (1996). Intestinal mutagenicity of two carcinogenic food mutagens in transgenic mice: 2-amino-l-methyl-6-phenylimidazo[4, 5-*b*]pyridine and amino(α)carboline. Carcinogenesis 17, 2259–2265. 10.1093/carcin/17.10.2259 8895498

[B104] Zhang X. He Q. Xiang H. Song S. Miao Z. Yang C. (2014). Rapid access to α-carbolines via a one-pot tandem reaction of α, β-unsaturated ketones with 2-nitrophenylacetonitrile and the anti-proliferative activities of the products. Org. Biomol. Chem. 12, 355–361. 10.1039/c3ob41921e 24263172

[B105] Zhu J. K. Gao J. M. Yang C. J. Shang X. F. Zhao Z. M. Lawoe R. K. (2020). Design, synthesis, and antifungal evaluation of neocryptolepine derivatives against phytopathogenic fungi. J. Agric. Food Chem. 68, 2306–2315. 10.1021/acs.jafc.9b06793 31995378

