# Supplementary material for: Comprehensive review of α-carboline alkaloids: Natural products, updated synthesis, and biological activities
Source: Front Chem. 2022 Aug 26;10:988327. doi: 10.3389/fchem.2022.988327 (PMC9459053; doi:10.3389/fchem.2022.988327)
Supplement: Supplementary file 1 [file DataSheet1.docx]

Supplementary Material

# Supplementary Schemes

**Scheme 1**. A unified approach to the isomeric α‑, β‑, γ‑, and δ‑Carbolines using a palladium-catelyzed Ullmann cross-coupling methods. (a) Cu, CuI, Pd(dppf)Cl_2_·CH_2_Cl_2_, DMSO, 50 ℃, 6.25 h, 82 %; (b) H_2_, 10 wt % Pd/C, MeOH, 22 ℃, 16 h, 75 %; (c) 10 wt % Pd/C, Ph_2_O, 210 ℃, 0.66 h, 97 %.

**Scheme 2.** Two methods to afford annulated 2-aryl-α-carboline heterocycles.

**Scheme 3.** Synthesis of dihydro-α-carboline and α‑carboline via annulation of pyridine ring methods.

## Supplementary Figures

**Supplementary Figure 1.** Structure and bioactivity of compound **83**.

**Supplementary Figure 2.** Structures and bioactivity of compounds **84-87**.

**Supplementary Figure 3.** Structures of α-carbolines **98-101** with anti-tumor ability.

**Supplementary Figure 4.** Structure and bioactivity of the representative 2,4-substituted α-carboline derivative **109**.

**Supplementary Figure 5.** Structure and anti-plasmodial activity of compound **117.**

**Supplementary Figure 6.** Structure and anti-microbial activity of the representative compound **118**.
